# LptO (PG0027) Is Required for Lipid A 1-Phosphatase Activity in Porphyromonas gingivalis W50

**DOI:** 10.1128/JB.00751-16

**Published:** 2017-05-09

**Authors:** Minnie Rangarajan, Joseph Aduse-Opoku, Ahmed Hashim, Graham McPhail, Zofia Luklinska, M. Florencia Haurat, Mario F. Feldman, Michael A. Curtis

**Affiliations:** a Institute of Dentistry, Barts and The London School of Medicine & Dentistry, Queen Mary University of London, London, United Kingdom; b Cellular Pathology, Barts Health NHS Trust, London, United Kingdom; c Nanovision Centre, Advanced Electron Microscopy, School of Engineering & Materials Science, Queen Mary University of London, London, United Kingdom; d Department of Biological Sciences, University of Alberta, Edmonton, Alberta, Canada; Michigan State University

**Keywords:** Porphyromonas gingivalis, PG0027, MALDI-TOF MS, MALDI-TOF/TOF MS, electron microscopy, phosphatase activity

## Abstract

Porphyromonas gingivalis produces outer membrane vesicles (OMVs) rich in virulence factors, including cysteine proteases and A-LPS, one of the two lipopolysaccharides (LPSs) produced by this organism. Previous studies had suggested that A-LPS and PG0027, an outer membrane (OM) protein, may be involved in OMV formation. Their roles in this process were examined by using W50 parent and the Δ*PG0027* mutant strains. Inactivation of *PG0027* caused a reduction in the yield of OMVs. Lipid A from cells and OMVs of P. gingivalis W50 and the Δ*PG0027* mutant strains were analyzed by matrix-assisted laser desorption ionization–time of flight mass spectrometry (MALDI-TOF MS). Lipid A from W50 cells contained bis-P-pentaacyl, mono-P-pentaacyl, mono-P-tetraacyl, non-P-pentaacyl, and non-P-tetraacyl species, whereas lipid A from Δ*PG0027* mutant cells contained only phosphorylated species; nonphosphorylated species were absent. MALDI-TOF/TOF tandem MS of mono-P-pentaacyl (*m*/*z* 1,688) and mono-P-tetraacyl (*m*/*z* 1,448) lipid A from Δ*PG0027* showed that both contained lipid A 1-phosphate, suggesting that the Δ*PG0027* mutant strain lacked lipid A 1-phosphatase activity. The total phosphatase activities in the W50 and the Δ*PG0027* mutant strains were similar, whereas the phosphatase activity in the periplasm of the Δ*PG0027* mutant was lower than that in W50, supporting a role for PG0027 in lipid A dephosphorylation. W50 OMVs were enriched in A-LPS, and its lipid A did not contain nonphosphorylated species, whereas lipid A from the Δ*PG0027* mutant (OMVs and cells) contained similar species. Thus, OMVs in P. gingivalis are apparently formed in regions of the OM enriched in A-LPS devoid of nonphosphorylated lipid A. Conversely, dephosphorylation of lipid A through a PG0027-dependent process is required for optimal formation of OMVs. Hence, the relative proportions of nonphosphorylated and phosphorylated lipid A appear to be crucial for OMV formation in this organism.

**IMPORTANCE** Gram-negative bacteria produce outer membrane vesicles (OMVs) by “blebbing” of the outer membrane (OM). OMVs can be used offensively as delivery systems for virulence factors and defensively to aid in the colonization of a host and in the survival of the bacterium in hostile environments. Earlier studies using the oral anaerobe Porphyromonas gingivalis as a model organism to study the mechanism of OMV formation suggested that the OM protein PG0027 and one of the two lipopolysaccharides (LPSs) synthesized by this organism, namely, A-LPS, played important roles in OMV formation. We suggest a novel mechanism of OMV formation in P. gingivalis involving dephosphorylation of lipid A of A-LPS controlled/regulated by PG0027, which causes destabilization of the OM, resulting in blebbing and generation of OMVs.

## INTRODUCTION

All of the Gram-negative bacteria studied so far have been shown to produce outer membrane vesicles (OMVs) via blebbing of the outer membrane (OM) that are then released as spheres ∼50 to 250 nm in diameter (1, 2) and secreted into the medium, and this process is highly conserved and is not confined to pathogens. OMVs possess multiple functional roles (3) and can reach targets that are far removed from the bacterial cell from which they originate. They can function offensively as delivery mechanisms for virulence factors such as toxins or proteases, as well as LPS (lipopolysaccharide), peptidoglycan (PG), and flagellin (4–7), and defensively to aid in the colonization of a host and survival of the organism in a hostile environment (2, 8).

The Gram-negative bacterial envelope consists of an OM, an inner membrane (IM), and the periplasm (a concentrated, gel-like matrix), which is found in the periplasmic space between the two membranes. LPS is the main component of the outer leaflet of the OM, and a layer of phospholipids is present in the inner leaflet of the OM. Thus, the outermost layer of the OMVs contains predominantly LPS and phospholipids. OMVs also contain integral OM and OM-anchored lipoproteins and periplasmic proteins. Evidence from studies of OMVs from several bacteria shows that OMV production is a regulated mechanism since some components of the OM and periplasm are present in OMVs in amounts different from those in the cell (4–6, 9–11).

Although the levels of OMV production in Gram-negative bacteria can be affected by environmental factors or mutations in certain genes, non-OMV-producing strains have not been isolated (2, 12), suggesting that vesiculation is an essential process.

The Gram-negative anaerobe Porphyromonas gingivalis is an important agent in the etiology of adult periodontal disease and produces several virulence factors that include extracellular cysteine proteases with specificities for Arg-X (Arg-gingipains, Rgps) and Lys-X (Lys-gingipain, Kgp) peptide bonds (13) and two LPSs, namely, O-LPS (14) and A-LPS (15, 16), that play important roles in the deregulation of innate and inflammatory systems in the host (17, 18). The O-PS repeating unit of O-LPS is composed of →3)-α-d-Gal*p*-(1→6)-α-d-Glc*p*-(1→4)-α-l-Rha-(1→3)-β-d-GalNAc*p*-(1→, and the A-PS repeating unit of A-LPS is built up of a phosphorylated branched d-Man-containing oligomer composed of an α1→6-linked d-mannose backbone to which α1→2-linked d-Man side chains of different lengths (one or two residues) are attached at position 2. One of the side chains contains Manα1→2Manα-1-phosphate linked via phosphorus to a backbone Man residue at position O-2. Previous studies of P. gingivalis W50 have shown that there is selective packaging of proteins and virulence factors into the OMVs of P. gingivalis and certain abundant OM proteins are excluded (11), and one of the two LPSs synthesized by this organism, namely, A-LPS, is directly involved in this sorting mechanism.

*PG0027* (*lptO*) encodes an integral OM protein that appears enriched in the OMVs of P. gingivalis W50. Ishiguro et al. (19) identified PG0027 as a unique membrane protein in P. gingivalis W83 and an essential component of a novel secretion pathway for gingipains, and Sato et al. (20) identified PG0027 as one of the components of the PorSS secretion system, also referred to as the type IX secretion system, in P. gingivalis. In this system, proteins that possess a characteristic CTD (C-terminal domain) are secreted into the extracellular milieu. Chen et al. (21) described a mutation in *PG0027* of P. gingivalis W50 and ATCC 33277 and suggested a role for LptO in the deacylation of monophosphorylated lipid A that was linked to the coordinated secretion of A-LPS and CTD proteins by a novel secretion-and-attachment system to form a structured surface layer.

In this paper, we describe the construction and characterization of a Δ*PG0027* deletion mutant of P. gingivalis W50 and the influence of this mutation on OMV formation. The results show that there are significant differences between P. gingivalis parent strain W50 and the Δ*PG0027* mutant strain with respect to OMV production. Furthermore, rather than an effect on deacylation, loss of PG0027 led to loss of dephosphorylation of monophosphoryl lipid A species. This led us to hypothesize that PG0027 has a role in OMV production in P. gingivalis by promoting lipid A dephosphorylation and thereby influencing the balance of phosphorylated and nonphosphorylated lipid A, which may serve to destabilize localized areas of the OM.

## RESULTS

### Characterization of P. gingivalis mutant strains.

**FIG 1:**
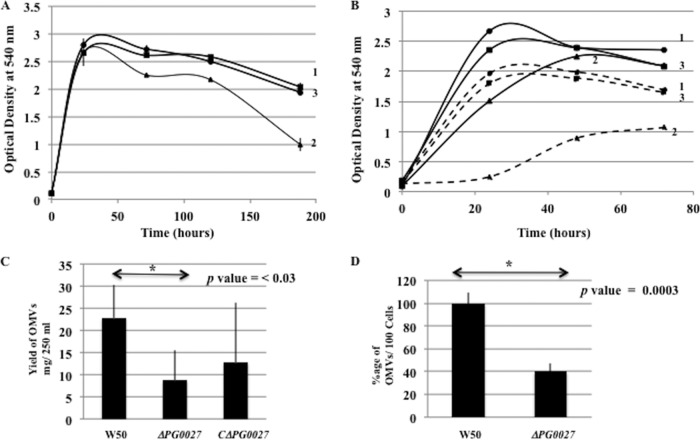
Properties of P. gingivalis W50, the Δ*PG0027* mutant strain, and the *C*Δ*PG0027* strain. (A) Growth in BHI broth. Samples were withdrawn at different time points, and the OD_540_ was measured for 8 days. Curves: 1, W50; 2, Δ*PG0027* mutant strain; 3, *C*Δ*PG0027* strain. (B) Strains were grown in BHI broth as for panel A (solid line) or with the addition of 0.02% Tween 20 (dashed line). Curves: 1, W50; 2, Δ*PG0027* mutant strain; 3, *C*Δ*PG0027* strain. (C) Histogram showing OMV yields from P. gingivalis W50 and the Δ*PG0027* mutant and *C*Δ*PG0027* strains. Student's *t* test yielded a *P* value of <0.05. (D) Counting of OMVs from TEM experiments. Counts of OMVs present in the Δ*PG0027* mutant strain are expressed as a percentage of the OMV counts present in W50 (100%) (*P* = 0.0003). Strains were grown in an anaerobic cabinet in BHI broth supplemented with hemin.

The growth of P. gingivalis strain W50 and that of the complemented Δ*PG0027* (*C*Δ*PG0027*) strain are very similar throughout the period tested (∼ 8 days), whereas the Δ*PG0027* mutant strain shows a decrease in optical density (OD) after 72 h, indicating cell lysis; a typical growth curve is shown in Fig. 1A. The Δ*PG0027* mutant strain is nonpigmenting (not shown). The growth curves of P. gingivalis W50 and the Δ*PG0027* and *C*Δ*PG0027* strains were determined in duplicate, and the error bars are shown in Fig. 1A. The Δ*PG0027* mutant strain was more susceptible to growth inhibition in the presence of Tween 20 (0.02%) than the parent and complemented strains (Fig. 1B), suggesting that the mutant is less robust and more sensitive to detergents.

### Electron microscopy.

P. gingivalis W50 and the Δ*PG0027* mutant strain were examined by scanning electron microscopy (SEM), and W50 and the Δ*PG0027* and *C*Δ*PG0027* strains were examined by transmission electron microscopy (TEM).

### SEM.

**FIG 2:**
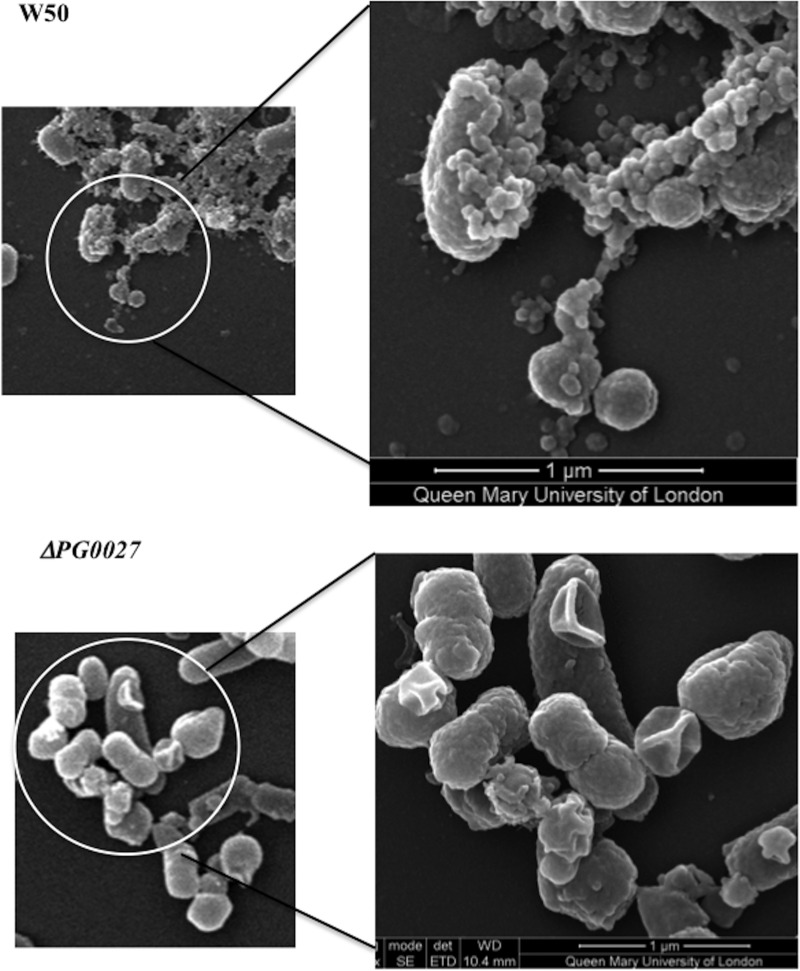
SEM of P. gingivalis W50 and the Δ*PG0027* mutant strain. Samples were prepared for SEM as described in Materials and Methods. The scale bar represents 1 μm. P. gingivalis W50 shows the characteristic membrane blebbing forming OMVs, which is not present in the Δ*PG0027* mutant strain.

Figure 2 shows the images obtained by SEM of P. gingivalis W50 and Δ*PG0027* mutant cells grown in brain heart infusion (BHI) broth for 24 h. P. gingivalis W50 (Fig. 2) shows a large number of OMVs covering the cell surface, whereas in the P. gingivalis Δ*PG0027* mutant strain, there are large perturbations of the cell surface rather than the characteristic 50- to 100-nm OMVs of parent strain W50, even though the bacterial cells used in these procedures were in late log/early stationary phase (Fig. 1A). Such atypical OM perturbations may relate to the susceptibility to detergents and to cell lysis in later stationary phase observed in this strain (Fig. 1B).

### TEM.

**FIG 3:**
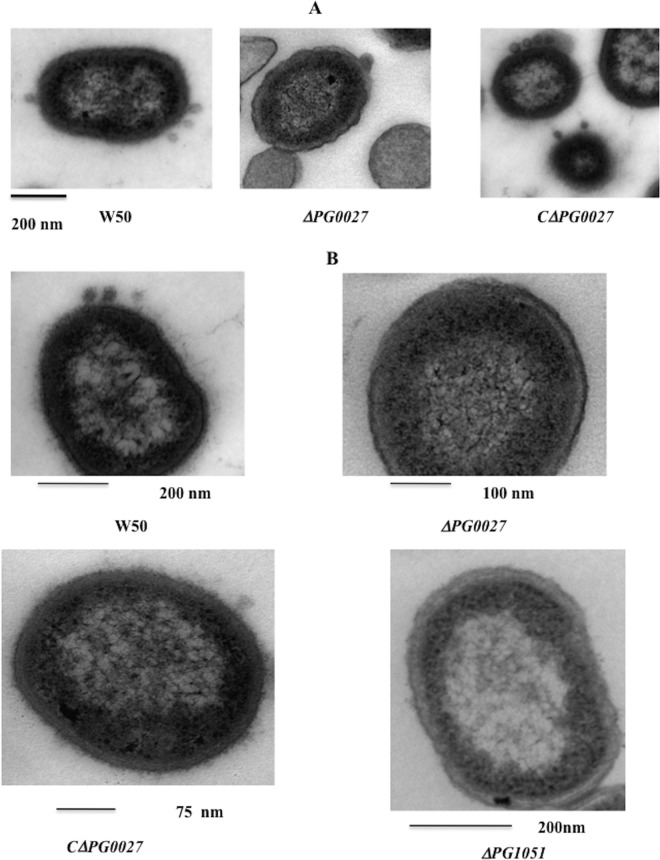
TEM of P. gingivalis W50, the Δ*PG0027* and Δ*PG1051* (*waaL*) mutant strains, and the *C*Δ*PG0027* strain. Samples were prepared for TEM as described in Materials and Methods. (A) The scale bar represents 200 nm. OMV formation in P. gingivalis W50 and the *C*Δ*PG0027* strain is clearly visible as defined structures. (B) Close-up view of the outer surface layers of P. gingivalis W50, the Δ*PG0027* and Δ*PG1051* (*waaL*) mutant strains, and the *C*Δ*PG0027* strain.

Representative images of P. gingivalis W50 and the Δ*PG0027* and *C*Δ*PG0027* strains obtained by TEM are shown in Fig. 3A. OMVs are seen blebbing off the OM in P. gingivalis W50 and the *C*Δ*PG0027* strain, whereas in the P. gingivalis Δ*PG0027* mutant strain, the cell surface shows some “ripples” and the characteristic OMV structures are absent. This reiterates the observations from SEM (Fig. 2) and the growth rate/lysis of the P. gingivalis Δ*PG0027* mutant strain (Fig. 1), suggesting that the OM of the mutant strain is altered.

The TEM images obtained from cells of P. gingivalis W50 and the *C*Δ*PG0027* strain show an electron-dense region around the surface of the cells (16) that is absent in the P. gingivalis Δ*PG0027* and Δ*PG1051* (*waaL*) mutant strains (Fig. 3B), which are shown for comparison.

### OMVs.

The yields of OMVs (milligrams per 250 ml of culture) obtained from P. gingivalis W50, the Δ*PG0027* mutant strain, and the *C*Δ*PG0027* strain are shown in Fig. 1C. While there was wide batch-to-batch variation in the yields of OMVs from an individual strain, there was a statistically significant reduction (by ∼50%) (*P* < 0.05) in the Δ*PG0027* mutant strain compared to parent strain W50. The *C*Δ*PG0027* strain showed an increase in the yield of OMVs compared to the Δ*PG0027* mutant strain, indicating that the trend was toward restoration of OMV formation by complementation. Low-resolution TEM images were subjected to visual inspection, and OMV formation was also determined by counting. The number of OMVs per 100 cells of the Δ*PG0027* mutant strain was significantly reduced to a similar extent (∼50%) (*P* < 0.003) as that determined by weight per culture volume (Fig. 1D) of parent strain W50.

### Gel electrophoresis of LPS.

Synthesis of both O-LPS and A-LPS appears to be unaffected in the Δ*PG0027* mutant strain, the *C*Δ*PG0027* strain, and the Δ*PG902*/*PG0027* and Δ*PG1711*/*PG0027* mutant strains (see Fig. S2 in the supplemental material).

### Analysis of lipid A in whole cells (OM) by MALDI-TOF MS.

**FIG 4:**
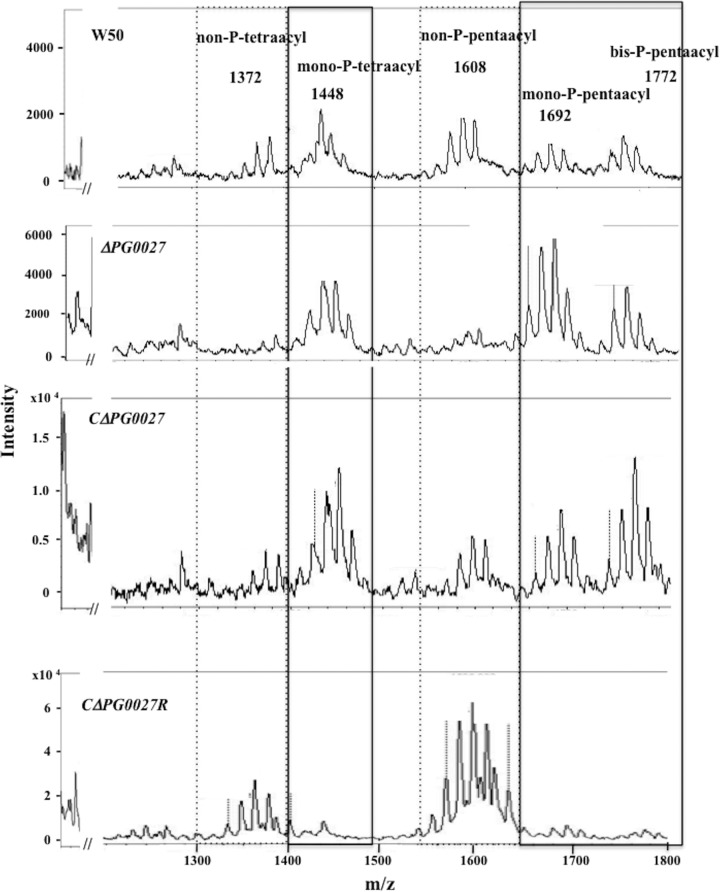
MALDI-TOF MS analysis of lipid A from P. gingivalis W50, the Δ*PG0027* mutant strain, and the *C*Δ*PG0027* and *C*Δ*PG0027R* strains. Negative-ion MALDI-TOF MS was performed on lipid A samples with norharmane as the matrix as described in Materials and Methods. Boxes with solid lines represent the mono-P-tetraacyl, mono-P-pentaacyl, and bis-P-pentaacyl lipid A clusters, whereas boxes with dashed lines represent the non-P-tetraacyl and non-P-pentaacyl lipid A clusters.

Since it has previously been suggested that PG0027 is a lipid A deacylase (21), we examined the lipid A composition of LPS in whole cells of P. gingivalis W50, the Δ*PG0027* mutant strain, and the *C*Δ*PG0027* and *C*Δ*PG0027R* strains by MALDI-TOF MS (Fig. 4). Average masses are used throughout. Lipid A from whole cells (W50) contained five main clusters of peaks detected at approximately *m*/*z* 1,772, *m*/*z* 1,692, *m*/*z* 1,608, *m*/*z* 1,448, and *m*/*z* 1,372 (Fig. 4). The clusters around *m*/*z* 1,772 could be assigned to bis-P-pentaacyl species, those around *m*/*z* 1,692 could be assigned to mono-P-pentaacyl species, those around *m*/*z* 1,608 could be assigned to non-P-pentaacyl species, those around *m*/*z* 1,448 could be assigned to mono-P-tetraacyl species, and those around *m*/*z* 1,372 could be assigned to non-P-tetraacyl species (Fig. 4). Kumada et al. (22) described the fatty acids present in the lipid A of P. gingivalis as two molecules of (R)-3-OH-i C_17:0_ and one each of (R)-C_16:0_, (R)-3-OH-C_16:0_, and (R)-3-OH-i C_15:0_. Heterogeneity within these clusters reflects differences in fatty acid chain length (14 mass units), a phenomenon previously ascribed to the relaxed specificity of the fatty acyl chain transferase activity of P. gingivalis (23). Conversely, lipid A from Δ*PG0027* only contained signals for bis-P-pentaacyl (*m*/*z* 1,772), mono-P-pentaacyl (*m*/*z* 1,692), and mono-P-tetraacyl (*m*/*z* 1,448) species; signals for non-P-pentaacyl (*m*/*z* 1,608) and non-P-tetraacyl (*m*/*z* 1,372) species were either very low or absent. Hence, rather than the reported loss of deacylation following inactivation of *PG0027*, these data suggest that PG0027 is required for lipid A dephosphorylation while the acylation status is unaltered by this mutation.

The lipid A of the *C*Δ*PG0027* strain showed clusters similar to those present in P. gingivalis W50 lipid A (Fig. 4). In the complemented strain using the *rgpA* promoter to induce expression (the *C*Δ*PG0027R* strain), the results were dramatically different (Fig. 4). In this case, only signals for nonphosphorylated lipid A species, namely, non-P-pentaacyl (*m*/*z* 1,608) and non-P-tetraacyl (*m*/*z* 1,372) species, were detected.

**FIG 5:**
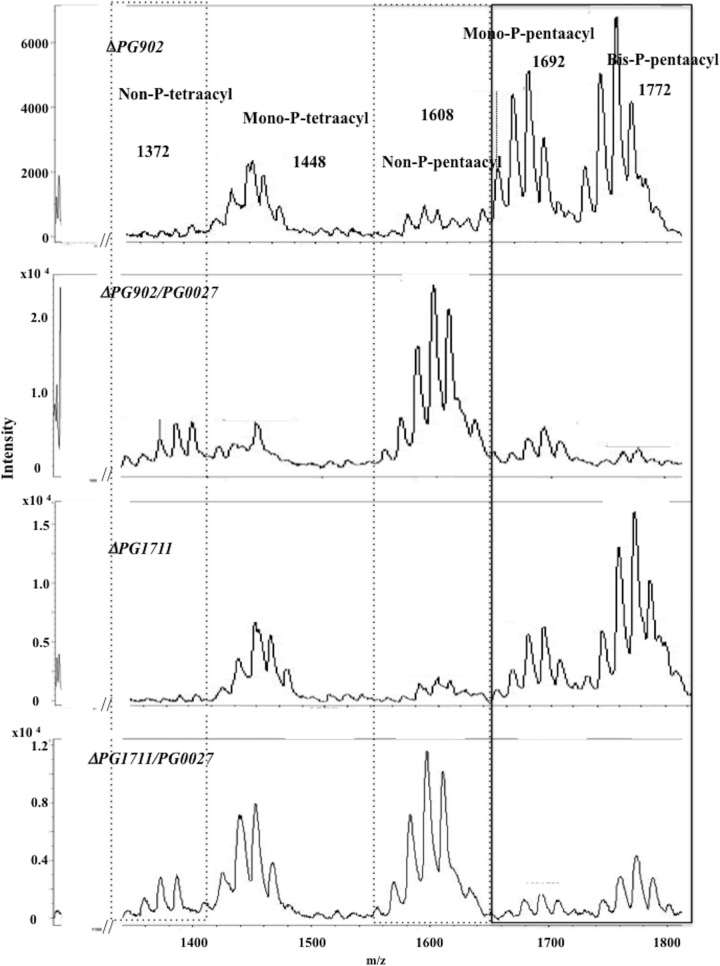
MALDI-TOF MS analysis of lipid A from the P. gingivalis Δ*PG902*, Δ*PG902*/*PG0027*, Δ*PG1711*, and Δ*PG1711*/*PG0027* mutant strains. Boxes with solid and dashed lines represent phosphorylated and nonphosphorylated species, respectively, as described in the legend to Fig. 4.

We also examined the gene dose effect by examining the effect of introducing a second copy of *PG0027* into two strains in which the α-mannosidase genes *PG902* and *PG1711* (24) had been inactivated, thereby providing a defined site for the insertion of an additional copy of *PG0027*. The lipid A isolated from Δ*PG902*/*PG0027* and Δ*PG1711*/*PG0027* mutant strains (with an extra copy of *PG0027* inserted) was also analyzed by MALDI-TOF MS (Fig. 5). Lipid A preparations from the P. gingivalis Δ*PG902* and Δ*PG1711* α-mannosidase mutant strains showed profiles similar to those from parent strain W50 when analyzed by MALDI-TOF MS, with major signals for bis-P-pentaacyl (*m*/*z* 1,772), mono-P-pentaacyl (*m*/*z* 1,692), and mono-P-tetraacyl (*m*/*z* 1,448) species (Fig. 5) and smaller signals for non-P-pentaacyl (*m*/*z* 1,608) and non-P-tetraacyl (*m*/*z* 1,372) species. However, lipid A from the P. gingivalis Δ*PG902*/*PG0027* and Δ*PG1711*/*PG0027* mutant strains showed a large increase in the signals for non-P-pentaacyl (*m*/*z* 1,608) species and a demonstrable increase in non-P-tetraacyl (*m*/*z* 1,372) species (Fig. 5) compared to the corresponding parent strains.

These results confirm that PG0027 plays an important role in the dephosphorylation of lipid A and do not support a role for this protein in lipid A deacylation. Complementation of the Δ*PG0027* mutation restores the lipid A profile to that seen in the P. gingivalis W50 parent strain, whereas in the complemented overexpressing *C*Δ*PG0027R* strain, only the dephosphorylated pentaacyl and dephosphorylated tetraacyl lipid A species are present. Furthermore, addition of a second copy of *PG0027* leads to higher levels of lipid A dephosphorylation. Thus, PG0027 appears to control/balance the levels of the five lipid A species normally present in P. gingivalis W50 (Fig. 4) by a direct or indirect effect on dephosphorylation of the phosphorylated species of lipid A.

### Analysis of lipid A isolated from OMVs.

**FIG 6:**
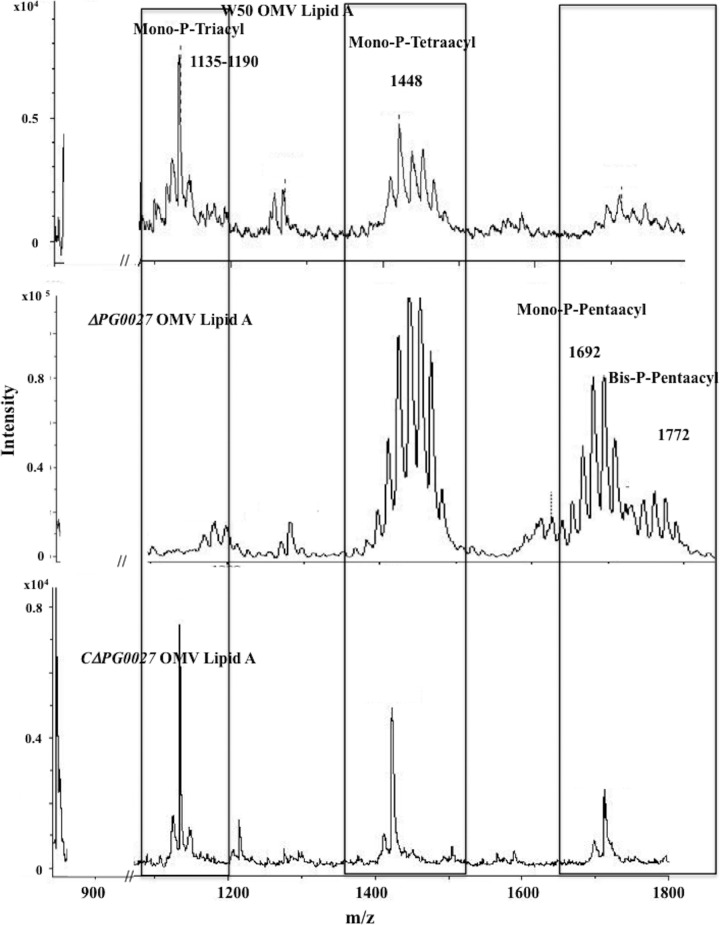
MALDI-TOF MS analysis of lipid A isolated from OMVs from P. gingivalis W50, the Δ*PG0027* mutant strain, and the *C*Δ*PG0027* strain. MALDI-TOF MS was performed in negative-ion mode as described in Materials and Methods. Boxes represent mono-P-triacyl, mono-P-tetraacyl, and phosphorylated-pentaacyl lipid A species.

We have previously shown extensive lipid A remodeling in the OMVs of P. gingivalis W50 (11). Lipid A samples isolated from OMVs of P. gingivalis W50 and the Δ*PG0027* and *C*Δ*PG0027* strains were analyzed by MALDI-TOF MS, and the spectra are shown in Fig. 6. The main signals observed by MALDI-TOF MS of OMVs (W50) are for the mono-P-tetraacyl (*m*/*z* 1,448) and mono-P-triacyl (*m*/*z* 1,135 to 1,190) species. Signals for the nonphosphorylated species were very low or absent (Fig. 6). Similarly, in the *C*Δ*PG0027* strain, the main signals observed for the lipid A from OMVs were for the mono-P-triacyl (*m*/*z* 1,135 to 1,190) and mono-P-tetraacyl species (Fig. 6) (although the signals were lower in intensity) and nonphosphorylated species were absent. Thus, in P. gingivalis W50 and the *C*Δ*PG0027* strain, it appears that OMVs do not contain any dephosphorylated lipid A species and are therefore formed in regions of the OM that are devoid of nonphosphorylated lipid A. In contrast, the MALDI-TOF MS profiles of lipid A isolated from the OMVs of the Δ*PG0027* mutant strain were almost identical to the profile of lipid A from whole cells of the Δ*PG0027* mutant strain (see Fig. 4).

### Analysis of lipid A during growth of P. gingivalis from 4 to 24 h.

There appears to be no increase in the amount of A-LPS synthesized between 4 and 24 h of growth, as determined by SDS-PAGE and Western blotting versus monoclonal antibody 1B5 titration (data not shown), whereas the dephosphorylation of lipid A species appears to be an active process that occurs between ∼8 h and 24 h of growth, as detected by MALDI-MS analysis (see Fig. S4).

### MALDI-TOF/TOF MS/MS of lipid A.

**FIG 7:**
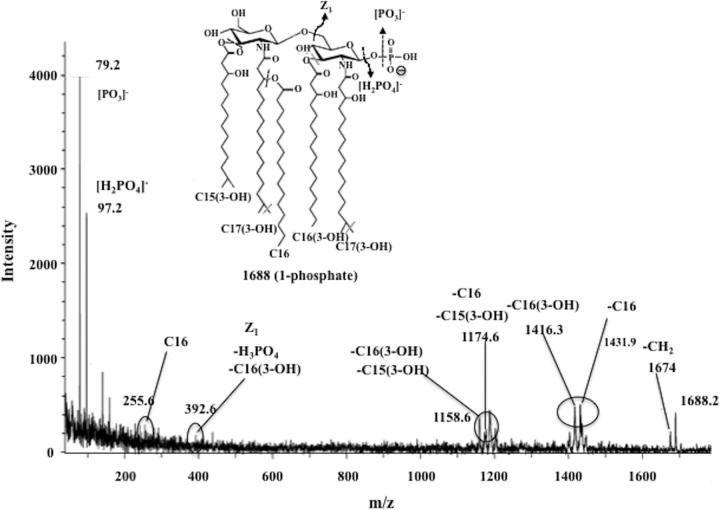
MALDI TOF/TOF tandem mass spectrum of *m*/*z* 1,688 of lipid A from the P. gingivalis Δ*PG0027* mutant strain. Inset structures show the proposed phosphate positioning, and dashed lines and arrows indicate possible cleavage sites.

**FIG 8:**
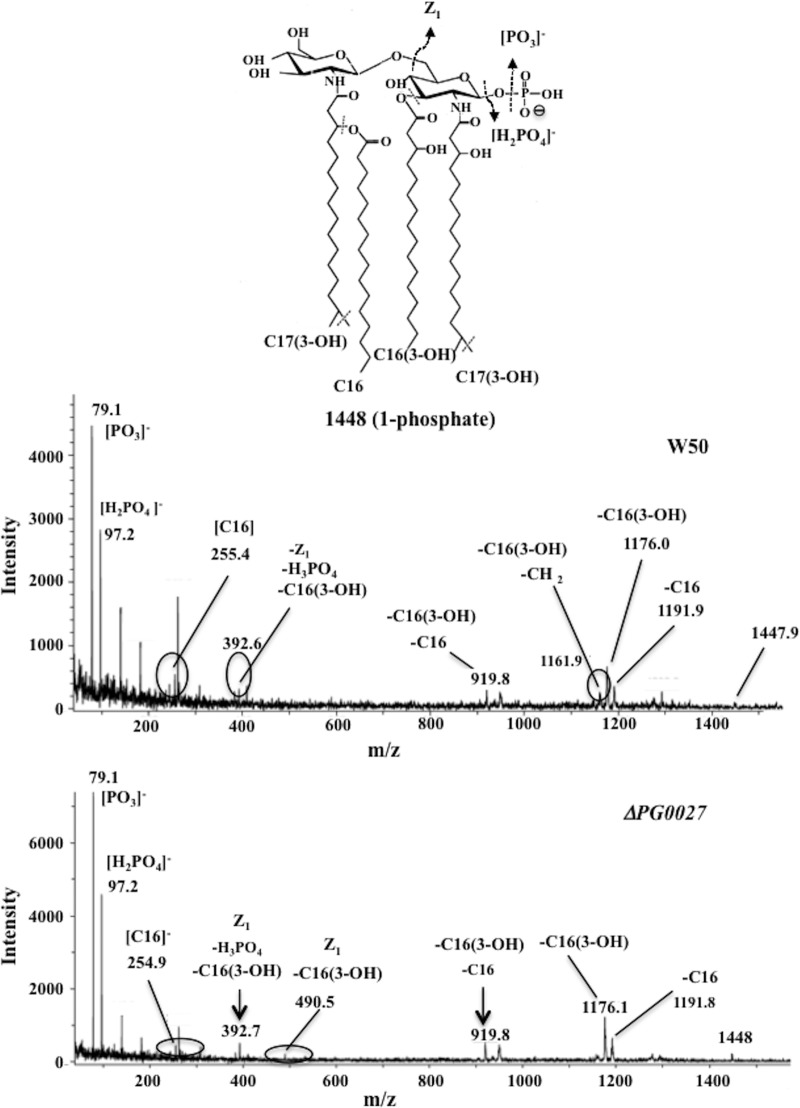
MALDI TOF/TOF tandem mass spectrum of *m*/*z* 1,448 of lipid A from P. gingivalis W50 (A) and the Δ*PG0027* mutant strain (B). Inset structures show the proposed phosphate positioning, and dashed lines and arrows indicate possible cleavage sites.

Since the profiles of the lipid A species of the P. gingivalis Δ*PG0027* mutant strain obtained by MALDI-TOF MS showed the absence of nonphosphorylated species, it was relevant to identify the position of the phosphate group in the mono-P-pentaacyl and mono-P-tetraacyl species of lipid A from the parent and mutant strains, i.e., whether it is a lipid A 1-phosphate or a lipid A 4′-phosphate. MALDI-TOF/TOF tandem MS (MS/MS) analyses of the lipid A cluster centered around the *m*/*z* 1,688 (mono-P-pentaacyl) and *m*/*z* 1,448 (mono-P-tetraacyl) species were performed. The results are shown in Fig. 7 and 8.

No cross-ring fragments were observed in the TOF/TOF MS/MS spectra of the lipid A structures of *m*/*z* 1,688 from the P. gingivalis Δ*PG0027* mutant strain (Fig. 7) and *m*/*z* 1,448 (Fig. 8) from both P. gingivalis W50 and the Δ*PG0027* mutant strain. Neutral losses of C-16, C-16(3-OH), C-15(3-OH), phosphoric acid, and various combinations of these were observed as product ions in the TOF/TOF MS/MS spectrum of the mono-P-pentaacyl species of lipid A (*m*/*z* 1,688) (Fig. 7). Since the mono-P-pentaacyl negative ions (*m*/*z* 1,688) were recovered in comparatively small amounts from P. gingivalis W50, it was not possible to perform MALDI-TOF/TOF MS/MS analysis of this peak. In the TOF/TOF MS/MS spectrum of the *m*/*z* 1,448 species of lipid A from P. gingivalis W50 and Δ*PG0027*, there were signals for the neutral losses of C-16(3-OH) and C-16 fatty acids, phosphoric acid, and fragment Z_1_ [lacking C-16(3-OH) and phosphoric acid] (Fig. 8) (25). Since there were no cross-ring fragments in the glucosamine (I), this indicates the presence of a phosphate group at C-1. Thus, the mono-P-pentaaacyl (*m*/*z* 1,688) and mono-P-tetraacyl (*m*/*z* 1,448) species of lipid A from both P. gingivalis W50 and the Δ*PG0027* mutant strain carry the phosphate at C-1. Thus, we conclude that the phosphatase deficient in the Δ*PG0027* mutant strain is lipid A 1-phosphatase.

### Phosphatase assays.

The results described so far have indicated that PG0027 has a role to play in the dephosphorylation of lipid A. Therefore, phosphatase activities in culture supernatants, intact whole cells, and sonicated supernatants of P. gingivalis W50, the Δ*PG0027* mutant strain, and the *C*Δ*PG0027* and *C*Δ*PG0027R* strains were measured with 4-nitrophenylphosphate as the substrate at 30°C at pHs between 7.4 and 8.3 in either discontinuous or continuous assays with a recording spectrophotometer. No phosphatase activity was present in the culture supernatants of P. gingivalis W50, the Δ*PG0027* mutant strain, or the *C*Δ*PG0027* and *C*Δ*PG0027R* strains, suggesting that this activity was not secreted into the culture medium by P. gingivalis.

**FIG 9:**
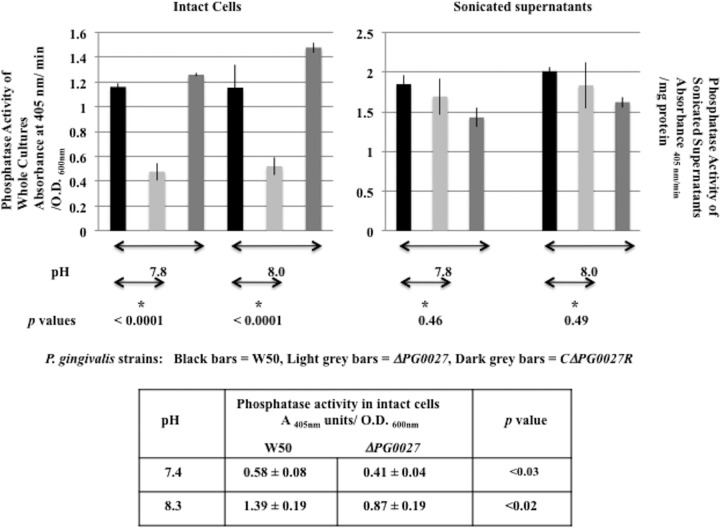
Phosphatase activities of intact cells and sonicated supernatants of P. gingivalis strains. Phosphatase activities were measured with 4-nitro-phenylphosphate as the substrate as described in Materials and Methods and expressed as units based on the change in the *A*_405_/OD_600_ ratio of intact cells and as units based on the change in the *A*_405_ per milligram of protein in sonicated supernatants. Activities were measured at pHs 7.4, 7.8, 8.0, and 8.3 with either continuous or discontinuous assays. The activities obtained at pHs 7.8 and 8.0 are shown as histograms. Black bars, P. gingivalis W50; light gray bars, Δ*PG0027*; dark gray bars, *C*Δ*PG0027R. P* values (Student's *t* test) are indicated below the pairs. The activities obtained at pHs 7.4 and 8.3 and the *P* values determined from the data are shown at the bottom.

Phosphatase activities in sonicated cell supernatants (which is a measure of total phosphatase activity) (periplasm plus membranes plus cytoplasm) of P. gingivalis W50 and the Δ*PG0027* mutant and *C*Δ*PG0027R* strains were measured at pHs 7.4 to 8.3, and activities at pHs 7.8 and 8.0 are shown in Fig. 9. The P. gingivalis Δ*PG0027* mutant strain contains ≥90% of the phosphatase activity present in parent strain W50, indicating that the phosphatase activities in the sonicated supernatants of P. gingivalis W50 and the Δ*PG0027* mutant strain are comparable.

Phosphatase activities in intact cells of P. gingivalis W50 and the Δ*PG0027* mutant strain grown for 24 h in BHI broth were measured with 4-nitrophenylphosphate concentrations of 0.25 to 4 mM, and the maximum velocities were determined with ENZFitter software. In this case, the assays are most likely a measure of the activities present in the periplasmic space/IMs. The phosphatase activities in intact cells of P. gingivalis W50 appear to show a pH maximum at 7.8 to 8.0, and the activities measured at these two pH values are shown (Fig. 9). It was not possible to measure the enzyme activities of intact cells at pHs >8.3, as alkaline buffers caused cell lysis. Enzyme activities present in the periplasm/IM of P. gingivalis W50 and the *C*Δ*PG0027R* strain are significantly higher in the pH range of 7.8 to 8.0 than the activities in the P. gingivalis Δ*PG0027* mutant strain, which varied between 40 and 50% of the activity present in parent strain W50 (Fig. 9).

The phosphatase activities of intact cells measured in these experiments are significantly lower in the Δ*PG0027* mutant strain than those in W50 (*P* < 0.0001 at pH 7.8 and *P* < 0.0001 at pH 8.0). At pHs 7.4 and 8.3, the phosphatase activity of intact cells of the Δ*PG0027* mutant strain were significantly lower (*P* < 0.03 at pH 7.4 and *P* < 0.02 at pH 8.3) than those of parent strain W50 (Fig. 9, bottom). Overall, these data indicate that there is only a slight reduction (∼90% of the activity of parent strain W50) in the total phosphatase production of the Δ*PG0027* mutant strain, but the localization of the activity or the presence of inhibitory factors is altered such that less activity resides in the periplasm/IM of this mutant strain. It is therefore possible that the reduced phosphatase activity in the periplasm/IM of the Δ*PG0027* mutant strain could be due to the absence of lipid A phosphatase activity, which would correlate with the lower levels of dephosphorylated lipid A in this mutant strain.

### Cloning and expression of *PG0027* in Escherichia coli.

**FIG 10:**
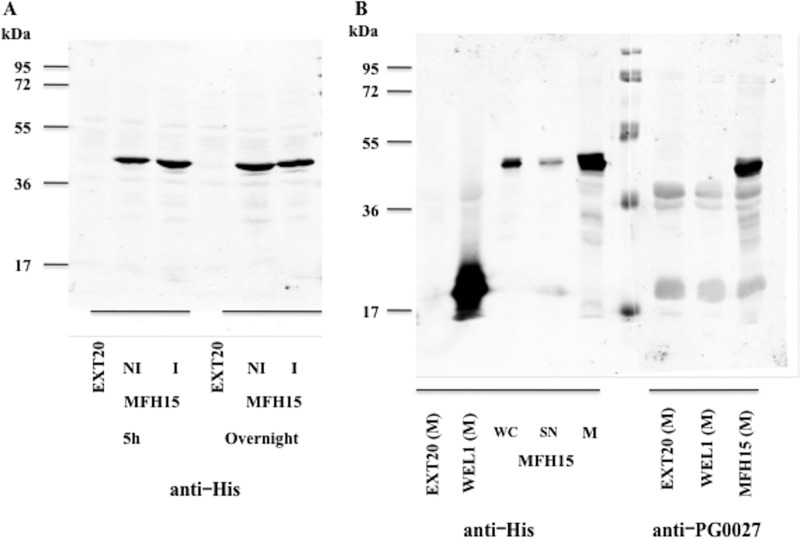
SDS-PAGE and Western blotting of proteins from E. coli DH5α cells containing pEXT20, pMFH15, and pWEL1 (*S*. Typhimurium PagL cloned into pEXT20) versus anti-His and anti-PG0027 antibodies. (A) Proteins from EXT20 and MFH15 (NI, noninduced; I, induced) for 5 h or overnight were subjected to SDS-PAGE and Western blotting and probed with an anti-His antibody. (B) SDS-PAGE and Western blotting of membranes from EXT20 (M) and WEL1 (M) and samples from MFH15, namely, whole cells (WC), supernatants (SN), and membranes (M) probed with an anti-His antibody. Membranes from EXT20 (M), WEL1 (M), and MFH15 (M) were also probed with an anti-PG0027 antibody.

Figure 10 shows the SDS-PAGE and Western blotting of proteins from E. coli DH5α cells containing pEXT20, pMFH15, and pWEL1 versus anti-His and anti-PG0027 antibodies. PG0027 is expressed and appears to be enriched in the membrane fraction.

### *In vitro* assays of PG0027 lipid A modification activity.

**FIG 11:**
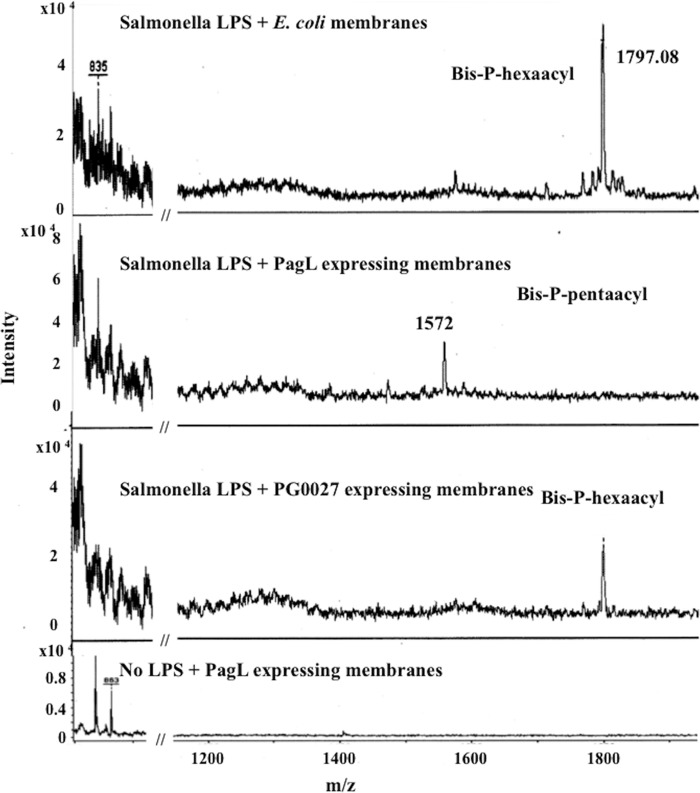
Lipid A-modifying activity of *S*. Typhimurium PagL and P. gingivalis PG0027 expressed in E. coli with Salmonella LPS as the substrate. Salmonella LPS was incubated with PagL-expressing membranes (in E. coli) and PG0027-expressing membranes (in E. coli), and lipid A was isolated from the reaction mixture and analyzed by MALDI-TOF MS in linear negative-ion mode.

**FIG 12:**
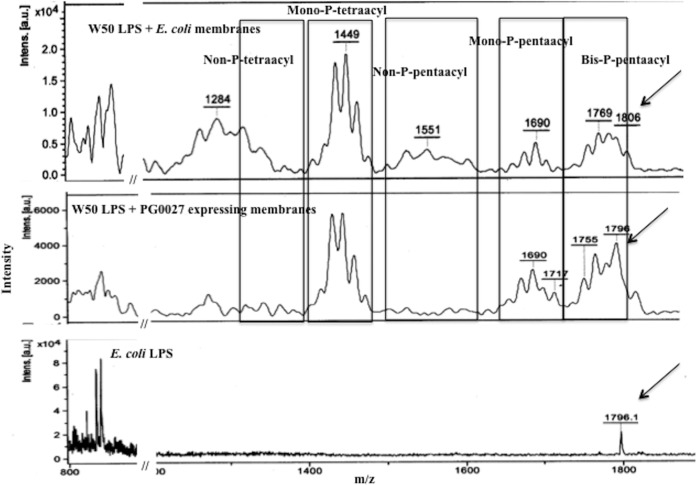
Lipid A modification assays of PG0027-expressing membranes (in E. coli) with P. gingivalis W50 LPS as the substrate. P. gingivalis W50 LPS was incubated with E. coli membranes containing EXT20 (top panel) or PG0027-expressing membranes (MFH15) (middle panel). Lipid A was isolated from the reaction mixture as described in Materials and Methods and analyzed by MALDI-TOF MS in linear negative-ion mode. The bottom panel shows lipid A isolated from E. coli DH5α cells. The arrows show E. coli lipid A. Intens. [a.u.], intensity in arbitrary units.

Deacylase activity of PagL (Salmonella enterica serovar Typhimurium) expressed in E. coli (26) was studied with *S*. Typhimurium LPS as the substrate, which served as a control to ensure that the assays were feasible. Membranes from E. coli expressing PG0027 were used in *in vitro* assays with the *S*. Typhimurium and P. gingivalis W50 LPSs as substrates as described in Materials and Methods. Lipid A was isolated from the reaction mixtures (27) and analyzed by MALDI-TOF MS, and the results are shown in Fig. 11 and 12. The MALDI-TOF MS spectrum of lipid A from *S*. Typhimurium LPS shows the presence of bis-P-hexaacyl species at *m*/*z* 1,798 (Fig. 11). After the exposure of *S*. Typhimurium LPS to membranes containing PagL expressed in E. coli, the MALDI-TOF MS spectrum of lipid A shows the disappearance of the bis-P-hexaacyl peak (*m*/*z* 1,798) and the appearance of a bis-P-pentaacyl-peak at *m*/*z* 1,572, indicating that PagL shows lipid A 3-*O*-deacylase activity. However, membranes containing PG0027 expressed in E. coli do not alter the MALDI-TOF MS spectrum of Salmonella lipid A (Fig. 11), suggesting that PG0027 does not (3-*O*-)deacylate or dephosphorylate Salmonella LPS.

MALDI-TOF MS of W50 lipid A isolated from a reaction mixture containing W50 LPS incubated with E. coli membranes (containing the vector alone) shows signals for bis-P-pentaacyl, mono-P-pentaacyl, non-P-pentaacyl, and mono-P-tetraacyl species at *m*/*z* 1,772, 1,689, 1,570, and 1,448, respectively, and bis-P-hexaacyl species from E. coli lipid A at *m*/*z* 1,796 (Fig. 12). After incubation with E. coli membranes expressing PG0027 and PagL (not shown), the MALDI-TOF MS spectrum does not show major changes (Fig. 12). There is no change in the acylation patterns of the clusters of the four lipid A species, suggesting that PG0027 does not possess LPS/lipid A deacylase or phosphatase activity.

## DISCUSSION

### Role of PG0027.

PG0027 is enriched in the OMVs of P. gingivalis W50 and a variety of mutant strains we have previously examined (11). In this work, we focused on the properties of PG0027 in P. gingivalis in relation to OMV formation. Production of OMVs in the Δ*PG0027* mutant strain was reduced by over 50%, and this was partially restored by complementation.

### How might the action of PG0027 contribute to OMV formation?

P. gingivalis PG0027 is a 43.3-kDa protein of 391 amino acids that lacks cysteine residues and possesses a typical signal sequence and a short, low-complexity peptide that is abundant in many proteins (28). Further analysis by protein modeling via secondary-structure prediction with PHYRE^2^ (protein homology recognition engine; http://www.sbg.bio.ic.ac.uk/phyre2/html/page.cgi?id=index) (29) identified PG0027 as an OM porin that probably transports fatty acids, as this is the same fold group as a long-chain fatty acid transporter (FadL, the highest score) of E. coli. FadL, a member of a conserved family of OM proteins involved in the uptake of hydrophobic compounds and aromatic hydrocarbons (30), is a monomer with a long (∼50-Å) barrel composed of 14 antiparallel β strands (more than other OM proteins) and does not contain a channel connecting the external environment of the cell with the periplasm. Chen et al. (21) described LptO as a deacylase because lipid A from an *lptO* mutant of P. gingivalis W50, when analyzed by MALDI-TOF MS in either positive- or negative-ion mode, was unable to produce mono-P-tetraacyl species of lipid A. We are unable to explain this inconsistency between the two studies, although it may relate to the method of lipid A preparation. The procedure we used to prepare lipid A yields clean preparations without the presence of phospholipids such as ceramides, which were reported by Chen et al. (21), and the MALDI-TOF MS spectra are therefore far easier to interpret. It should also be noted that the method of lipid A preparation used in the present work is the standard methodology employed by most groups in the field. Furthermore, the use of these methods allowed us to be the first to identify the presence of non-P lipid A in this organism (16, 31), and this has subsequently been confirmed by other research groups (17, 32). The use of norharmane as the matrix in MALDI-TOF MS allows us to observe both nonphosphorylated and phosphorylated lipid A species in negative-ion mode and thus avoid the need to switch instrument polarity. The profiles of lipid A from P. gingivalis W50 and the Δ*PG0027* mutant strain obtained by MALDI-TOF MS showed that PG0027 had a role in the dephosphorylation of lipid A because of the absence of non-P lipid A species in the latter. The lower levels of phosphatase activity in the periplasm of the Δ*PG0027* mutant strain is consistent with this observation, and MALDI-TOF/TOF MS/MS of mono-P-pentaacyl and mono-P-tetraacyl species of lipid A present in the LPS of the Δ*PG0027* mutant strain suggests that it is the lipid A 1-phosphatase activity that is deficient in this strain.

### Could P. gingivalis PG0027 be a lipid A phosphatase?

The crystal structures of several alkaline phosphatases from bacterial (33–36) and human (37, 38) sources indicate that the enzyme is a homodimer containing three metal-binding sites per subunit. The enzyme contains two domains, the larger core domain, mostly from the N-terminal region, is composed of a central eight- or nine-stranded β-sheet that is sandwiched between two sets of helices of various lengths, eight on one side and four on the other side of the sheet. The β-sheet is parallel, except for one antiparallel strand in the middle. The C-terminal crown domain sits on top of the molecule and consists of flexible loops containing short α-helices on the surface and is formed by the insertion of a 60-residue segment from each monomer. The catalytic sites are at the interface between the two subunits, contain two binding sites for Zn^2+^ and one for Mg^2+^, and are exposed to the environment. On that basis, it is therefore unlikely that PG0027 is a lipid A 1-phosphatase because its structure and location in the OM are not consistent with previous studies of this group of enzymes. Conversely, Hoopman et al. (39) have described an acid phosphatase (MapA, 105 kDa) from the Gram-negative commensal organism Moraxella catarrhalis that is an OM transporter. MapA has a 23-amino-acid signal sequence, and the N-terminal portion (amino acids 24 to 279) shows similarity to class A bacterial nonspecific acid phosphatases (NSAPs) (39) and shows about 23% identity over this region to the NSAP of Escherichia blattae, including the active-site His at position 233. The latter is a compact all-α-helical protein, and its crystal structure has been solved (40). The CTD (amino acid residues 672 to 940) is homologous to the β domains of conventional autotransporters and probably forms a β-barrel structure with a 12-β-stranded transmembrane pore with an α-helix occupying the center of the pore on the basis of available crystal structures (39). However, it is unlikely that PG0027 has properties similar to those of MapA because it is a much smaller protein essentially comprising only the β-barrel domain.

To experimentally address whether PG0027 acts as a lipid A 1-phosphatase as described above, we performed activity assays with recombinant PG0027 expressed in E. coli by using the PagL deacylase of Salmonella expressed in E. coli as a control. PG0027 did not exhibit either lipid A *O*-deacylase or phosphatase activity against W50 lipid A or Salmonella lipid A, in contrast to the complete deacylation of Salmonella bis-P-hexa-acylated lipid A by PagL, in the assays. While we cannot rule out the possibility that the recombinant PG0027 protein was expressed in an inactive form, these data, together with the structural information given earlier, strongly suggest that PG0027 is not a lipid A-modifying enzyme. This suggests that PG0027 has an indirect effect on the lipid A 1-phosphatase activity in P. gingivalis. Thus, (i) lipid A 1-phosphatase activity in the P. gingivalis Δ*PG0027* mutant strain is synthesized but not transported into the periplasm, (ii) the lipid A 1-phosphatase enzyme is synthesized and transported into the periplasmic space but is unable to hydrolyze the lipid A 1-phosphate because of the presence of a potent inhibitor/regulator of enzyme activity, or (iii) the lipid A 1-phosphatase enzyme is not synthesized in the Δ*PG0027* mutant strain. The similar levels of total cellular phosphatase activity but differential levels in the periplasm in the parent and mutant strains suggest that the first two of these options are the most likely. Therefore, it is possible that PG0027 acts as a transporter or as an inhibitor/regulator of periplasmic lipid A 1-phosphatase activity in P. gingivalis W50.

The numbers and identities of lipid A phosphatases in P. gingivalis are still controversial. Coats et al. (17) described the contribution of phosphatases in the hemin-induced remodeling of P. gingivalis lipid A structures. By performing BLAST searches comparing amino acid sequence of Francisella novicida LpxE against that of P. gingivalis W83 (41), Coats et al. (17) identified PG1587 and PG1773 as the highest scoring proteins belonging to the PAP2 phosphatase superfamily, which included known lipid A phosphatases (42). However, analysis of lipid A isolated from single- and double-knockout mutants has not resolved the identity/nature of all lipid A phosphatases (17, 43). In a more recent study, Zenobia et al. (43) concluded that a PG1587 knockout strain accumulated the bis-P-pentaacyl lipid A species and a PG1773 knockout strain accumulated the mono-1-P-tetraacyl lipid A species. However, these results do not explain why the remaining phosphate group in their lipid A from ΔPG1587 has not been removed by the action of the functional PG1773 lipid A 1-phosphatase.

### Lipid A phosphorylation and OM stability.

The phosphorylation status of lipid A will influence the stability of the OM and also the potential formation of OMV. One of the unusual features of the OM of Gram-negative bacteria is the asymmetric distribution of lipids in the inner (phospholipids) and outer (LPS) faces. The outer face of the OM can interact with cations in the milieu (44) because LPS contains more charge per unit of surface area than any phospholipid and is anionic at neutral pH because of the exposed phosphate groups (44). LPS shows a high state of order and forms a relatively rigid structure, mainly because of the rigid lipid A (45, 46), as determined by X-ray powder diffraction, molecular modeling, and conformational energy calculations, whereas the O-specific chain was found to be the most flexible and could be stretched to significant distances into the extracellular space, which could explain why antibodies binding to O-specific chains at distances of >20 nm can be seen in electron microscopy studies (47).

Atomic force microscopy (AFM), used as a pressure-measuring device, has shown that for the isolated B-band LPS of Pseudomonas aeruginosa, resistance cannot be detected until the tip of the AFM is ∼10 nm from the surface of an LPS micelle (48), although freeze substitution has shown that in P. aeruginosa PAO1, the longer chain and electronegative B band of LPS can extend up to 40 nm from the OM (44, 49). To solve this discrepancy, several B-band LPS molecules of P. aeruginosa were dynamically modeled by using brush theory as they interact with one another on the surface of the OM. This study showed that O side chains were in constant motion, such that only a proportion of the O chains was in the extended conformation at any given time, and these were probably captured by the rapid freezing used in the freeze substitution technique (49). Thus, although A-LPS contains negatively charged A-PS side chains, metal ion salt bridges between A-PS repeating units are not energetically possible, except at very high salt concentrations. Thus, the phosphate residues of lipid A are extremely important not only for binding to cationic antimicrobial peptides but also for binding to metal ions.

The presence of nonphosphorylated species of lipid A in the OM may be beneficial to the organism by enabling it to avoid immune detection through Toll-like receptor 4 binding (17, 32) and clearance and to avoid binding to positively charged cationic antimicrobial peptides, which can cause the formation of pores, resulting in cell lysis and cell death (50, 51).

The main nonbeneficial consequence of lipid A dephosphorylation, and hence the absence of negatively charged residues, is liable to be an inability to bind to divalent cations (which usually intercalate between the negatively charged phosphorylated lipid A species). Thus, the inability to bind divalent cations can potentially destabilize the OM. The absence of nonphosphorylated lipid A species, as in the case of the P. gingivalis Δ*PG0027* mutant strain, could stabilize the interactions between divalent cations and lipid A in the OM. Hence, lipid A dephosphorylation in P. gingivalis W50 is a regulated process caused by the ability of PG0027 to control the levels of phosphorylated and nonphosphorylated species of lipid A in A-LPS in the OM. The balance of these species has the potential to stabilize/destabilize the OM and hence influence the production of OMVs in this organism.

## MATERIALS AND METHODS

Chemicals were purchased from VWR, Lutterworth, Leicestershire, United Kingdom, or Sigma-Aldrich Co. Ltd., Poole, Dorset, United Kingdom, and were the purest grades available. Restriction and modification enzymes were purchased from New England BioLabs, and DNA purification reagents were obtained from Qiagen.

### Bacterial growth conditions.

P. gingivalis W50 and mutant strains were grown on blood agar plates containing 5% defibrinated horse blood or in BHI broth supplemented with hemin (5 μg ml^−1^) in an anaerobic atmosphere of 80% N_2_, 10% H_2_, and 10% CO_2_ (Don Whitely Scientific). Clindamycin (5 μg/ml) or tetracycline (1 μg/ml) was added when required. E. coli XL-1 Blue or XL-10 Gold (Stratagene) was used for plasmid maintenance and cloning. Antibiotics were added for cell selection (tetracycline HCl, 20 μg/ml) and plasmid selection (ampicillin [Na^+^ salt; 100 μg/ml] or erythromycin [300 μg/ml]).

### Construction of bacterial strains.

Construction and complementation of P. gingivalis mutant strains were performed as described previously (11) and are detailed in Table S1 and Fig. S1 in the supplemental material. The P. gingivalis Δ*PG902* and Δ*PG1711* mutant strains have been described elsewhere (24). Growth of P. gingivalis W50 and the Δ*PG0027* mutant and *C*Δ*PG0027* strains in BHI broth supplemented with hemin (5 μg/ml) was monitored over a period of 8 days by measuring the OD at 540 nm (OD_540_).

The susceptibility of P. gingivalis W50 and the Δ*PG0027* mutant strain to Tween 20 was performed as follows. Appropriate amounts of 2% Tween 20 stock solution were added to BHI broth (10 ml) to yield BHI broth containing 0, 0.005, 0.01, and 0.02% Tween 20. One milliliter of cultures of P. gingivalis strains grown for 24 h was used as the inoculum. The OD_540_ was measured every 24 h.

### Phosphatase assays.

Alkaline phosphatase activity was measured in continuous assays at pHs 7.8, 8.0, and 8.3 (in 0.25 M Tris-HCl buffer containing 5 mM MgCl_2_) at 30°C. The assay consisted of 0.8 ml of buffer and 0.2 ml of 4-nitrophenylphosphate (ranging in concentration from 20 to 1.25 mM such that the final concentration in the assay solution ranged from 4 to 0.25 mM). Reactions were initiated by the addition of 50 μl of whole cultures (intact cells) or sonicated supernatants of P. gingivalis W50, the Δ*PG0027* mutant, and the *C*Δ*PG0027* and *C*Δ*PG0027R* strains, and the increase in absorbance was measured with a Beckman DU 800 spectrophotometer. The rates obtained were corrected for ionization of the 4-nitrophenol product when performing continuous assays. Phosphatase activities were also measured at pHs 7.4, 7.8, and 8.0 with 4-nitrophenylphosphate as the substrate at 30°C in a discontinuous assay. Reaction mixtures contained 2 ml of buffer and 0.5 ml of 4-nitrophenylphosphate (concentrations ranging from 0.25 to 4 mM in the final reaction mixture) incubated at 30°C. The reaction was initiated by the addition of 50 μl of intact cells or sonicated supernatants of P. gingivalis W50 and the mutant and complemented strains. Aliquots (0.25 ml) were withdrawn at desired time intervals into 1-ml disposable cuvettes containing 0.75 ml of 0.75 M NaOH and mixed well, and the *A*_405_ was measured in a Beckman DU 800 spectrophotometer (Beckman Coulter) within 10 min. Each assay was done in triplicate, and mean values were used in calculations. Measurements of phosphatase activities in intact cells and sonicated supernatants were performed in triplicate as described above. Phosphatase activity was expressed as the change in *A*_405_ per minute per OD_600_ unit for intact cells or the change in *A*_405_ per milligram of protein for sonicated supernatants. Maximum rates and *K_s_* (substrate dissociation constant) values were obtained with ENZFITTER software (Biosoft, Cambridge, United Kingdom). Standard deviations were calculated by using the values measured for each set of experiments (four sets of experiments at each pH value, each individual assay performed in triplicate, and mean value used) and are indicated in Fig. 9.

### Sonicated supernatants for enzyme assays.

Cultures of P. gingivalis W50 and mutant strains grown for 24 h in BHI broth were centrifuged in Eppendorf tubes at 17,000 × *g* for 15 min at 4°C. The cells were washed once with 10 mM Tris-HCl (pH 7.3)–0.9% NaCl containing a complete cocktail of protease inhibitors (Roche) (buffer plus inhibitors). The washed cells were resuspended in a total of 2 ml of buffer plus inhibitors and sonicated with a small probe in a Soniprep Sonicator at a 12-μ amplitude for 5 × 30 s on ice. The sonicated samples were centrifuged in an Eppendorf centrifuge at 17,000 × *g* for 5 min at 4°C, and the supernatant was used in the measurement of phosphatase activities. Protein concentration was measured with the Bradford reagent (Bio-Rad) and bovine serum albumin as the standard.

### Isolation of lipid A.

Lipid A was isolated from freeze-dried whole bacterial cells (10 mg) and OMVs (5 to 10 mg) with TRIzol (Invitrogen) as described previously (27) and analyzed by MALDI-TOF MS. Lipid A was washed with 70 μl of ultrapure water and centrifuged in an Eppendorf centrifuge at 17,000 × *g* for 10 min at 22°C. Washing of lipid A enabled us to improve the quality of the MALDI-TOF spectra. The washed pellet remaining in the original tube was resuspended in 50 μl of ultrapure water and subjected to MALDI-TOF MS and gave clean spectra.

### MALDI-TOF MS.

MALDI-TOF MS was performed with a Bruker Microflex instrument fitted with a nitrogen laser operating at 337 nm with pulsed extraction in negative linear mode. Lipid A was analyzed with norharmane (9H-pyrido[3,4]indole) at a concentration of 10 mg/ml in methanol-water (2:1, vol/vol) as the matrix as previously described (16).

### MALDI-TOF/TOF MS/MS.

MALDI-TOF/TOF MS/MS was performed with an Autoflex TOF/TOF II (Bruker Daltonics Ltd.) fitted with a nitrogen laser operating at 337 nm. Full-scale MS scan negative ions were analyzed in reflectron mode with norharmane (10 mg/ml in water plus 0.1% trifluoroacetic acid) as the matrix. The instrument was externally mass calibrated. For MS/MS, negative precursor ions were analyzed by LIFT and data were recorded with flexControl version 3.0 and processed with flexAnalysis version 3.0 at Durham University (Mass Spectrometry Service, Department of Chemistry, Durham University, United Kingdom).

### Preparation of OMVs.

Volumes of 250 ml of cultures of P. gingivalis W50 and mutant strains grown in BHI broth for 24 h were centrifuged (Sorvall RC5C, SS34 rotor) at 26,000 × *g* and 4°C for 40 min, and the supernatant was filtered with a Nalgene 0.22-μm filtration apparatus. The filtrate was subjected to ultracentrifugation (Sorvall Discovery 100SE, T865 fixed-angle rotor) at 180,000 × *g* and 8°C for 1 h. The pellet was resuspended in sterile phosphate-buffered saline, and the ultracentrifugation step was repeated, followed by resuspension of the pellet in water and freeze-drying. The yield of OMVs was taken as the dry weight in milligrams per 250 ml of culture.

### Electron microscopy.

Cultures of P. gingivalis W50, the Δ*PG0027* mutant strain, and the *C*Δ*PG0027* strain grown in BHI broth for 24 h were centrifuged in an Eppendorf centrifuge at 17,000 × *g* and 4°C for 15 min, and the cell pellets were used.

### TEM.

Specimens were examined in a JEOL JEM1230 electron microscope (JEOL [UK] Ltd., JEOL House, Silver Court, Watchmead, Welwyn Garden City, United Kingdom), and digital images were captured with an Olympus Morada camera.

### SEM.

Imaging was performed with an FEI “Inspect” field emission scanning electron microscope (FEI Company, FEI Europe B.V., Eindhoven, The Netherlands), and secondary electron images were taken at 20 kV at a working distance of 10 mm.

### Statistical analysis.

A Student *t* test for paired values was used, and data were considered to be significant at a *P* value of <0.05.

## Supplementary Material

Supplemental material
