# Supplementary material for: LptO (PG0027) Is Required for Lipid A 1-Phosphatase Activity in Porphyromonas gingivalis W50
Source: J Bacteriol. 2017 May 9;199(11):e00751-16. doi: 10.1128/JB.00751-16 (PMC5424252; doi:10.1128/JB.00751-16)
Supplement: Supplemental material [file supp_199_11_e00751-16__index.html]

LptO (PG0027) Is Required for Lipid A 1-Phosphatase Activity in Porphyromonas gingivalis W50 — Supplemental material 

# LptO (PG0027) Is Required for Lipid A 1-Phosphatase Activity in Porphyromonas gingivalis W50

## Supplemental material

- Supplemental file 1 -

  Supplemental text; Fig. S1 (Construction of *P. gingivalis* Δ*PG0027* mutant strain), S2 (LPS from *P. gingivalis* strains), S3 (A-LPS in whole cells and OMVs of *P. gingivalis* W50), and S4 (MALDI-TOF MS analysis of lipid A from *P. gingivalis* W50); and Table S1 (Strains)

  PDF, 400K
