# Supplementary material for: LptO (PG0027) Is Required for Lipid A 1-Phosphatase Activity in Porphyromonas gingivalis W50
Source: J Bacteriol. 2017 May 9;199(11):e00751-16. doi: 10.1128/JB.00751-16 (PMC5424252; doi:10.1128/JB.00751-16)
Supplement: Supplemental material [file JB.00751-16_zjb999094405s1.pdf]

SUPPLEMENTAL MATERIAL.

Materials. PlusOne urea was purchased from GE Healthcare, Amersham Place, Buckinghamshire, U.K. A solution containing 30% acrylamide-Bis (37.5 : 1) was obtained from Bio-Rad Laboratories (Hercules, CA, USA). Horseradish peroxidase-labelled mouse immunoglobulin was purchased from DAKO A/S, High Wycombe, Buckinghamshire, U. K. cOmplete Mini Protease inhibitor cocktail tablets were obtained from Roche, Welwyn Garden City, Hertfordshire, U. K. Image J software (NIH, USA) was used to analyse Western blots.

Methods (Extended protocols).

Construction of *ΔPG0027* mutant

Generation of *ΔPG0027* followed a similar protocol to that described by Haurat *et al*. (1) (Table S1), by PCR amplification, DNA ligation, electroporation and colony screening. These procedures resulted in replacement of an internal 250bp of the 1,176bp PG0027 gene with the *erm* cassette (1) possessing stop codons and *rho*-independent transcription terminators to avoid read through. These are illustrated in Figure S1.

Construction of complemented strain of *P. gingivalis*

The procedures for DNA manipulation and construction of plasmids in *Escherichia coli* XL-1 Blue or XL-10 GOLD (Stratagene) have been previously described (2). For complementation of *ΔPG0027* mutant with a functional copy of *PG0027*, an amplicon corresponding to *PG0027* (1,173bp) preceded by an 827bp "regulatory unit" and 143bp downstream sequences, was cloned into pUCET1 (2) at BglII-NotI sites to generate pExp272. The plasmid was linearised with XbaI and transformed into *PG0027* to tetracycline resistance (1μg /ml). One representative complemented strain was selected (*CΔPG0027*) (Figure S1). Similarly, pExp272 was used to

deliver an extra copy of *PG0027* into the *erm* cassette within the mannosidase mutants *ΔPG0902* and *ΔPG1711* (Table S1).

Expression of PG0027 under *rgpA* promoter in *P. gingivalis ΔPG0027* mutant strain.

An amplicon corresponding to a 749bp DNA sequence 5' to the *rgpA* coding region and additionally possessing a NotI restriction site at the 5'-end, and a sequence at the 3'-end which overlapped with the 5'-end of *PG0027,* was generated by PCR with primers RgpApF1 and RgpApc27R1. Similarly, the coding region for *PG0027* (ATG [methionine] to the TAA [termination codon] to include a BamHI restriction site at the 3'-end, was separately amplified with primers PG0027F3 and PG0027R3. The two amplicons were mixed, heat-denatured and following slow annealing were used as targets in another PCR reaction with primers RgpApF1 and PG0027R3 to fuse the products together. The new product was digested with BamHI and NotI and cloned into the BglII-NotI site of pUCET1 (2) for subsequent integration into its cognate location in *P. gingivalis ΔPG0027* mutant by electroporation and selection for tetracycline resistance. Constructs were also monitored by DNA sequence and re-amplification of intended targets using appropriate combinations of primers. These processes generated a *P. gingivalis PG0027* complemented strain (*CΔPG0027R*) where the expression of the gene is under an *rgpA* promoter.

Cloning and expression of *PG0027* in *E. coli*

PG0027 was amplified from *P. gingivalis* W50 genomic DNA using the primers PG0027SacIfw(*aaaa***gagctc**ATGATTATCAAGAAAATGCTGAAAAATAAATTG) and PG0027-10HisHindIIIrv (*aaaa***aagctt**ttagtggtggtggtggtggtggtggtggtgGTGGAACAAATTGCGCAATCCATCC), irrelevant sequences are in italic, SacI and HindIII sites are in bold and the sequence encoding

the His_{10} tag is underlined. The PCR product and the plasmid pEXT20 (3) were digested with SacI and HindIII. Ligation of the restriction products resulted in the plasmid pMFH15, encoding a PG0027 with a C-terminal His_{10} tag. The plasmid was verified by DNA sequencing. The expression of pMFH15 was carried out in *E. coli* DH5α cells. Cells were grown at 37°C until they reached an OD_{600} of 0.4-0.5, induced with 0.5 mM IPTG and samples were withdrawn after 5h and overnight induction. The expression of PG0027-10His was verified by Western-blot analysis using either anti-His_6 polyclonal antibody (Rockland Immunochemicals Inc.) or anti-PG0027 polyclonal antibody (SACRI Antibody Services, University of Calgary, Canada). After incubation with a secondary goat anti-rabbit IRDye-680RD antibody (LI-COR Biosciences), the blots were scanned with an Odyssey infrared imaging system (LI-COR Biosciences).

Generation of anti- PG0027

PG0027Δ1-51 encoding *PG0027* without the signal peptide sequence was amplified from *P. gingivalis* W50 genomic DNA using the primers PG0027NdeIfw (*aaaa***catatg**GGGGATATAGGTGTGGC) and PG0027XhoI (*aaaa***ctcgag**GTGGAACAAATTGCGCAATCC), irrelevant sequences are italicized; NdeI and HindIII sites are in bold. The PCR product and the plasmid pET28a were digested with NdeI and XhoI. Ligation of the restriction products resulted in the plasmid pMFH9, encoding a truncated *PG0027* with an N-terminal His_6 tag. The plasmid was verified by DNA sequencing. The expression of the plasmid in *E. coli* BL21(DE3) was verified by Western-blot analysis using anti-His_6 polyclonal antibody (Rockland Immunochemicals Inc.). The strain was grown at 37°C to an OD_{600} around 0.3 and protein expression was induced with 0.1 mM IPTG and cells were harvested after 3 h by centrifugation at 10,000 x g for 10 min at 4°C. The pellets were gently resuspended in 20 mM Tris-HCl pH 8.0, 500 mM NaCl, 10mM imidazole, containing complete

EDTA-free protease inhibitor mixture (Roche Applied Science) and then lysed by sonication. The recombinant protein was purified from the inclusion bodies (IB) as described by Margetts *et al.* (4). Briefly, the IB were collected from the cell lysates by centrifugation at 20,000 x g for 10 min at 4°C, resuspended in binding buffer (20 mM Tris-HCl pH 8.0, 500 mM NaCl, 10mM imidazole, 6 M urea) and solubilized by rolling for 3 h at 4°C. Any insoluble material was removed by centrifugation. The supernatant containing the solubilized IB was loaded on a 1 ml HisTrap FF column (GE Healthcare) and the column was washed with 10 column volumes of binding buffer followed by 20 volumes of washing buffer (20 mM Tris-HCl pH 8.0, 500 mM NaCl, 60 mM imidazole, 6 M urea). The recombinant proteins were eluted with buffer (20 mM Tris-HCl pH 8.0, 500 mM NaCl, 250 mM imidazole, 6 M urea). The purified protein was dialysed exhaustively versus PBS. Rabbit polyclonal antibodies were raised against the recombinant protein at SACRI Antibody Services, University of Calgary, Canada.

Preparation of membranes

Membranes for use in *in vitro* lipid A modification/deacylation assays were prepared as described by Trent *et al* (5). Briefly, 100-ml cultures of *E. coli* DH5α cells containing the plasmids pEXT20 (3), pMFH15 or pWEL1 (*Salmonella typhimurium* PagL cloned into pEXT20 (6) were grown to an OD_{600} of ~0.4 at 37°C and 0.1 mM IPTG was added to induce protein expression. The cells were grown to an OD_{600} of 1 at 37°C and harvested by centrifugation at 7,000 × **g** for 15 min. All steps were carried out at 4°C. Cell pellets were resuspended in 50 mm Hepes, pH 7.5 supplemented with protein inhibitor (1 mM PMSF) and subjected to sonication. The crude lysate was cleared by centrifugation at 7,000 × g for 10 min. Membranes were prepared by two centrifugation steps at 100,000 × **g** for 60 min with a wash of the crude membranes in 50 mm Hepes, pH 7.5, after the first centrifugation to ensure the removal of all

cytosolic components. The final membrane pellet was resuspended in 50 mm Hepes, pH 7.5, 1mM PMSF. All samples were stored in aliquots at −80°C, and protein concentrations were measured using a Quick Start Bradford dye reagent and a BSA protein standard kit (Biorad), following the manufacturer's instructions.

LPS purification.

LPS for use in *in vitro* assays was purified by the procedure of Yi & Hackett (7). The 3-*O*-deacylase activity was assayed in a 15-μl reaction mixture containing 50 mm Hepes, pH 7.5, 0.1% Triton X-100, 0.5m NaCl, LPS purified from 10 mg of either *P. gingivalis* W50 or *S. typhimurium* cells and membrane preparation (final protein concentration 0.1 mg ml^{-1}). Reaction tubes were incubated at 30°C overnight and samples were dried by SpeedVac which stopped the reaction. Lipid A was purified as described previously (7) and MALDI MS was performed on a Bruker Daltonics (Bremen, Germany) UltrafleXtreme MALDI TOF/TOF mass spectrometer in linear negative mode.

Gel Electrophoresis.

SDS-PAGE and Western Blotting.

LPS from *P. gingivalis* W50, mutant *ΔPG0027*, complemented strains *CΔPG0027* and *CΔPG0027R*, strains *ΔPG902/PG0027* and *ΔPG1711/PG0027* were subjected to SDS-urea-PAGE at 10ºC for 3 to 4 h using procedures described previously by Inzana & Apicella (8). Gels were stained with silver using a commercial silver staining kit (Sigma, Poole, Dorset, U.K.). Samples were transferred onto nitrocellulose membranes and probed with MAb 1B5 or MAb 1A1 as described previously (9).

Transmission Electron Microscopy (TEM).

Specimens for negative staining were resuspended in distilled water and ~50 μl of suspension was pipetted onto pioloform coated grids. After 30 s, the excess was blotted off, stained with 2.5% phosphotungstic acid at pH 6.2 for 30 s and allowed to dry in air. Specimens for sectioning were fixed in phosphate buffered 4% glutaraldehyde and centrifuged at 500 x g for 10 minutes. The supernatant was removed and the pellet resuspended at 80ºC in 2% low temperature agarose. After allowing the gel blocks to set at room temperature, they were dissected into 1 mm cubes washed overnight in phosphate buffer. They were post-fixed in 1% aqueous osmium tetroxide, dehydrated in graded ethanol and embedded in Araldite. For electron microscopy, ultrathin sections (60 to 90 nm) were cut and stained with saturated aqueous uranyl acetate and Reynold's lead citrate

Scanning Electron Microscopy (SEM).

Specimens were spun down onto a microscope slide in a Shandon "Cytospin 3" cytocentrifuge and fixed in phosphate buffered 4% glutaraldehyde. After a buffer wash, they were post-fixed in 1% aqueous osmium tetroxide and dehydrated through graded ethanol. They were cleared with hexamethyldisilazane and allowed to dry in air before being mounted on SEM stubs and sputter coated with gold.

Titration of Cells and OMVs for A-LPS.

Freeze-dried whole cells and OMVs of *P. gingivalis* W50 were resuspended in 0.2% SDS and incubated at 22ºC for 30 minutes followed by the addition of 20 μl of Leupeptin (2 mg/ ml) and incubation for a further 10 minutes followed by heating at 100ºC for 5 minutes. The final concentration of whole cells and OMVs was 4 mg /ml. For titration of A-LPS, aliquots containing 0.2, 0.4, 0.6, 0.8, 1 and 1.2 μg of whole cells and OMVs were treated with SDS-sample buffer and subjected to SDS-PAGE using 12.5% acrylamide gels as described previously

by Laemmli(10). Loading control samples contained 0.8 μg of *P. gingivalis* W50 LPS. Samples were transferred onto nitrocellulose membranes and probed with MAb 1B5 as described previously (9). To ensure that equal amounts of W50 whole cell extracts and OMVs were loaded on the gels, the adhesin domains of HRgpA which are present in similar amounts in both samples (data not shown) were detected after SDS-PAGE and Western Blotting versus MAb 1A1 which cross reacts with adhesin domains (11). Quantitation of Western blots were performed using Image J software. The titration experiments were performed twice and the mean values were used.

Results.

Gel Electrophoresis of LPS.

P. gingivalis strains W50, *ΔPG0027*, *CΔPG0027*, *ΔPG902/PG0027* and *ΔPG1712/PG0027* produce LPS (predominantly O-LPS) seen on SDS-urea-PAGE (8) and silver staining of gels (Figure S2A) and the laddering patterns are identical. Western blotting versus MAb 1B5 (Figure S2B) shows that all the samples are also cross-reactive indicating that all the strains synthesise A-LPS. Thus, the synthesis of both O- and A-LPS appear to be unaffected in *ΔPG0027*, the complemented strain *CΔPG0027* and in strains *ΔPG902/PG0027* and *ΔPG1711/PG0027*.

Titration of Cells and OMVs for A-LPS.

The ratio of A-LPS in whole cells of *P. gingivalis* W50 to that in W50 OMVs is 1 : 3.8 ± 0.2 as analysed by Image J software (Figure S3) suggesting that A-LPS is enriched in OMVs of W50.

Since LPS gives a laddering pattern in SDS-urea-PAGE, quantitation of Western blots by Image J software analysis is not as straightforward as analysing single bands in protein blots. However, the results obtained above show that there appears to be selective enrichment of A-LPS in the OMVs obtained from the parent W50 strain.

Analysis of Lipid A during growth.

Analysis of lipid A during growth of *P. gingivalis* from 4h to 24h.

Lipid A was prepared from whole cells (OM) of *P. gingivalis* W50 grown in BHI for 4h, 8h and 24h and characterised by MALDI-TOF MS and the results are shown in Figure S4. Clusters of bis-P-pentaacyl-(m/z 1772), mono-P-pentaacyl- (m/z 1692) and mono-P-tetraacyl-(m/z 1448) lipid A species are present throughout the duration of the experiment whereas clusters of the non-P-pentaacyl- (m/z 1608) and non-P-tetraacyl- species (m/z 1372) appear after ~8h of growth and increase in amounts during growth up to 24h. Titration of A-LPS in *P. gingivalis* W50 cells by SDS-PAGE and Western Blotting vs MAb 1B5 during growth in BHI for 4h, 8h and 24h indicated that A-LPS was present in identical amounts in the cells at all the time points tested (data not shown).

Phosphatase Activity Measurements.

Modifications to lipid A including dephosphorylation normally occur in the periplasm/inner membrane fraction in Gram-negative bacteria (12-14). Previous studies of periplasmic phosphatase activity in *E. coli* and *C. difficile* have employed whole cell assays using a high concentration of substrate 4-nitrophenyl-phosphate (10 mM) in buffers containing 0.1% SDS and chloroform (15, 16) or in Tris-HCl buffers pH 8.8 as described for a marine Pseudomonad (17). However, inclusion of 0.1% SDS and chloroform in the assay buffer was deleterious to the

phosphatase activity present in *P. gingivalis* and hence the enzyme activities were measured in Tris-HCl buffers at pHs 7.8 and 8.0 without the addition of either 0.1% SDS or chloroform. Inclusion of SDS and chloroform in the assay buffer is not strictly necessary since the alkaline phosphatase is periplasmic (15) but it was used to ensure sterility of the assay tubes during long incubations.

***In vitro* assays of lipid A-modifying activity PG0027-expressed in *E. coli*.**

Deacylase activity of Pagl (*S. typhimurium*) expressed in *E. coli* (5) was studied using *S. typhimurium* LPS as substrate, which served as a control, to ensure that the assays were feasible. Membranes from *E. coli* expressing PG0027 were used in *in vitro* assays using *S. typhimurium* LPS and *P. gingivalis* W50 LPS as substrates as described in Methods. Lipid A was isolated from the reaction mixtures (7) and analysed by MALDI-TOF MS.

MALDI-TOF/TOF tandem MS of Lipid A.

Monophosphorylated lipid A containing 4'-phosphate and a free reducing terminus at position C-1 undergoes A-type cross-ring fragments ($^{0,2}A_2$ and $^{0,4}A_2$) in the reducing sugar A_2 (numbering of sugar residues from the non-reducing end) according to the nomenclature outlined by Domon & Costello (18) in the TOF/TOF tandem MS spectrum which are diagnostic for this species (19). Silipo *et al* (20) established that in the MS/MS of bis-phosphorylated doubly charged lipid A ions, (a) the loss of *O*-linked primary fatty acids takes place via two different mechanisms yielding two ions (which differ in mass by 18Da) described as "ketene residue loss" and "fatty acid residue" loss, (b) the loss of secondary fatty acids occurs only via a β-elimination mechanism (charge-remote fragmentation) leading to the loss of neutral fatty acids and (c) there is no loss of amide-linked fatty acids in lipid A. However, the loss of the primary fatty acid at position 3 was not observed or not assumed in the study by Silipo *et al* (20) as highlighted by

Kilar *et al* (21). This implies that the 3'-linked acyl- or acyloxyacyl ester moiety of a lipid A molecule with a 4' phosphate group can clearly be identified by the observation of which fatty acid has been eliminated with the result of two ions with an 18 Da difference in the mass spectrum (21).

REFERENCES.

1. **Haurat MF, Aduse-Opoku J, Rangarajan M, Dorobantu L, Gray MR, Curtis MA, Feldman MF.** 2011. Selective sorting of cargo proteins into bacterial membrane vesicles. J Biol Chem **286:**1269-1276.
2. **Rangarajan M, Aduse-Opoku J, Hashim A, Paramonov N, Curtis MA.** 2013. Characterization of the α- and β-Mannosidases of *Porphyromonas gingivalis*. J Bacteriol **195:**5297-5307.
3. **Dykxhoorn DM, St Pierre R, Linn T.** 1996. A set of compatible tac promoter expression vectors. Gene **177:**133-136.
4. **Margetts MB, Barr IG, Webb EA.** 2000. Overexpression, purification, and refolding of a *Porphyromonas gingivalis* cysteine protease from *Escherichia coli*. Protein Expr Purif **18:**262-268.
5. **Trent MS, Pabich W, Raetz CR, Miller SI.** 2001. A PhoP/PhoQ-induced Lipase (PagL) that catalyzes 3-O-deacylation of lipid A precursors in membranes of *Salmonella typhimurium*. J Biol Chem **276:**9083-9092.
6. **Elhenawy W, Bording-Jorgensen M, Valguarnera E, Haurat MF, Wine E, Feldman MF.** 2016. LPS Remodeling Triggers Formation of Outer Membrane Vesicles in Salmonella. MBio **7**.
7. **Yi EC, Hackett M.** 2000. Rapid isolation method for lipopolysaccharide and lipid A from gram-negative bacteria. Analyst **125:**651-656.
8. **Inzana T, Apicella M.** 1999. Use of a bilayer stacking gel to improve resolution of lipopolysaccharides and lipooligosaccharides in polyacrylamide gels. Electrophoresis **20:**462-465.
9. **Paramonov N, Rangarajan M, Hashim A, Gallagher A, Aduse-Opoku J, Slaney JM, Hounsell E, Curtis MA.** 2005. Structural analysis of a novel anionic polysaccharide from *Porphyromonas gingivalis* strain W50 related to Arg-gingipain glycans. Mol Microbiol **58:**847-863.
10. **Laemmli UK.** 1970. Cleavage of structural proteins during the assembly of the head of bacteriophage T4. Nature **227:**680-685.
11. **Curtis MA, Aduse-Opoku J, Slaney JM, Rangarajan M, Booth V, Cridland J, Shepherd P.** 1996. Characterization of an adherence and antigenic determinant of the ArgI protease of *Porphyromonas gingivalis* which is present on multiple gene products. Infect Immun **64:**2532-2539.
12. **Price NP, Jeyaretnam B, Carlson RW, Kadrmas JL, Raetz CR, Brozek KA.** 1995. Lipid A biosynthesis in *Rhizobium leguminosarum*: role of a 2-keto-3-deoxyoctulosonate-activated 4' phosphatase. Proc Natl Acad Sci U S A **92:**7352-7356.

13. **Wang X, Karbarz MJ, McGrath SC, Cotter RJ, Raetz CR.** 2004. MsbA transporter-dependent lipid A 1-dephosphorylation on the periplasmic surface of the inner membrane: topography of *Francisella novicida* LpxE expressed in *Escherichia coli*. J Biol Chem **279:**49470-49478.
14. **Wang E, Koutsioulis D, Leiros HK, Andersen OA, Bouriotis V, Hough E, Heikinheimo P.** 2007. Crystal structure of alkaline phosphatase from the Antarctic bacterium TAB5. J Mol Biol **366:**1318-1331.
15. **Berlin D.** 2010. In vivo analysis of protein translocation to the *Escherichia coli* periplasm, p 103 - 106. *In* Economou E (ed), Protein Secretion, Methods in Molecular Biology, vol 619. Springer Science + Business Media, LLC, Tolow, NJ, USA.
16. **Edwards AN, Pascual RA, Childress KO, Nawrocki KL, Woods EC, McBride SM.** 2015. An alkaline phosphatase reporter for use in *Clostridium difficile*. Anaerobe **32:**98-104.
17. **Thompson LMM, MacLeod RA.** 1974. Factors Affecting the Activity and Stability of Alkaline Phosphatase in a Marine Pseudomonad. Journal of Bacteriology **117:**813-818.
18. **Domon B, Costello C.** 1988. A Systematic nomenclature for carbohydrate fragmentations in FAB-MS/MS spectra of glycoconjugates. Glycoconjugate Journal **5:**397-409.
19. **Costello CE, Vath JE.** 1990. Tandem mass spectrometry of glycolipids. Methods Enzymol **193:**738-768.
20. **Silipo A, De Castro C, Lanzetta R, Molinaro A, Parrilli M, Vago G, Sturiale L, Messina A, Garozzo D.** 2008. Structural characterizations of lipids A by MS/MS of doubly charged ions on a hybrid linear ion trap/orbitrap mass spectrometer. J Mass Spectrom **43:**478-484.
21. **Kilár A, Dörnyei Á, Kocsis B.** 2013. Structural characterization of bacterial lipopolysaccharides with mass spectrometry and on- and off-line separation techniques. Mass Spectrom Rev **32:**90-117.

Figure S1. Construction of *P. gingivalis* mutant strain *ΔPG0027*.

A. Genetic organization of the PG0027 locus with arrows depicting relative positions, direction and sizes of Open Reading Frames (ORF). Oligonucleotide primers used in PCR reactions are indicated above ORF. B. For complementation, the area of the locus represented by primer pairs PG0027EF2/ PG0027ER2 was cloned into plasmid pUCET1 resulting in the fragment being tagged with *tetQ* and flanked by *ermF'* (non-functional *ermF*) and *ermAM*. This is used to target the *erm* cassette in the original mutant by homologous recombination as well as introducing *tetQ* marker into the genome of the complemented strain. C. Similar to B except the placement of *rgpA* promoter at the 5'-end of PG0027 ORF prior to complementation.

Figure S2. SDS-urea-PAGE of LPS from *P. gingivalis* strains followed by silver staining (A) and Western blotting vs MAb 1B5 (B).

(A): silver staining. (B): Western blotting vs MAb 1B5. Lanes: 1= W50, 2 = *ΔPG0027*(1), 3 = *ΔPG0027*(2), 4 = *CΔPG0027*, 5 = *ΔPG902/PG0027*, 6 = *ΔPG1711/PG0027*.

Figure S3. Titration of A-LPS in whole cells and OMVs of *P. gingivalis* W50 by SDS-PAGE and Western blotting vs MAb 1B5.

Whole cell extracts and OMVs of *P. gingivalis* W50 and containing 0.2, 0.4, 0.6, 0.8, 1 and 1.2 μg of material was subjected to SDS-PAGE and Western blotting vs MAb 1B5. Only the loading for 0.8 μg is shown. For loading controls, whole cell extracts and OMVs of *P. gingivalis* containing 4 μg of material was subjected to SDS-PAGE followed by Western blotting and probed with MAb 1A1. Images were scanned using Image J software.

Figure S4. MALDI-TOF MS analysis of lipid A from *P. gingivalis* W50 grown for 4 h, 8 h and 24 h.

Lipid A was isolated from *P. gingivalis* W50 cells grown for 4 h, 8 h and 24 h in BHI broth as described in Methods and MALDI-TOF MS was performed in the negative-ion mode using Norharmane as the matrix. Boxes with solid lines represent non-P-tetraacyl- and non-P-pentaacyl- lipid A species.

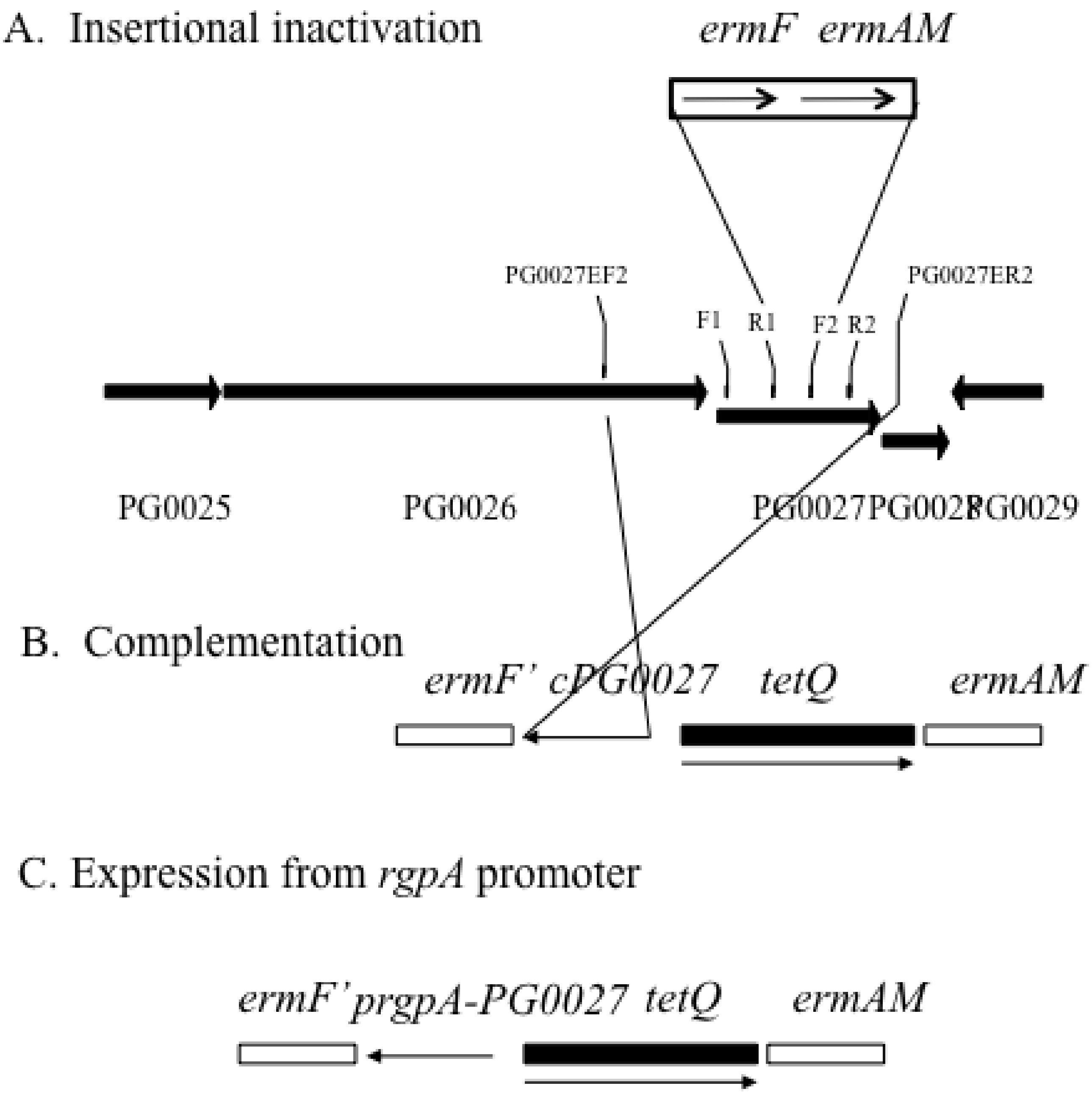

Figure S1. Construction of *P. gingivalis* mutant strain *ΔPG0027*. A. Genetic organization of the PG0027 locus with arrows depicting relative positions, direction and sizes of Open Reading Frames (ORF). Oligonucleotide primers used in PCR reactions are indicated above ORF. B. For complementation, the area of the locus represented by primer pairs PG0027EF2/PG0027ER2 was cloned into plasmid pUCET1 resulting in the fragment being tagged with *tetQ* and flanked by *ermF*' (non-functional *ermF*) and *ermAM*. This is used to target the *erm* cassette in the original mutant by homologous recombination as well as introducing *tetQ* marker into the genome of the complemented strain. C. Similar to B except the placement of *rgpA* promoter at the 5'-end of PG0027ORF prior to complementation.

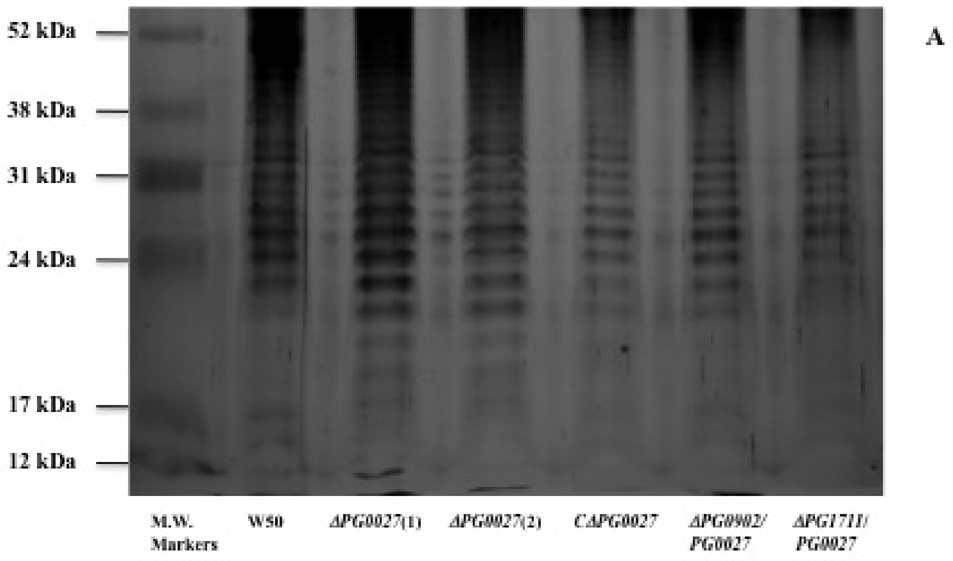

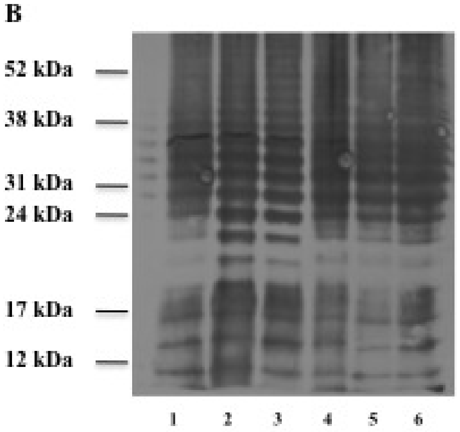

Figure S2. **SDS-Urea PAGE of LPS from *P. gingivalis* strains followed by silver staining (A) and Western blotting vs MAb 1B5 (B).**

Lanes: 1= W50, 2 =*ΔPG0027*(1), 3 = *ΔPG0027*(2), 4 = *CΔPG0027*, 5 =*ΔPG902/0027*, 6 = *ΔPG1711/0027*.

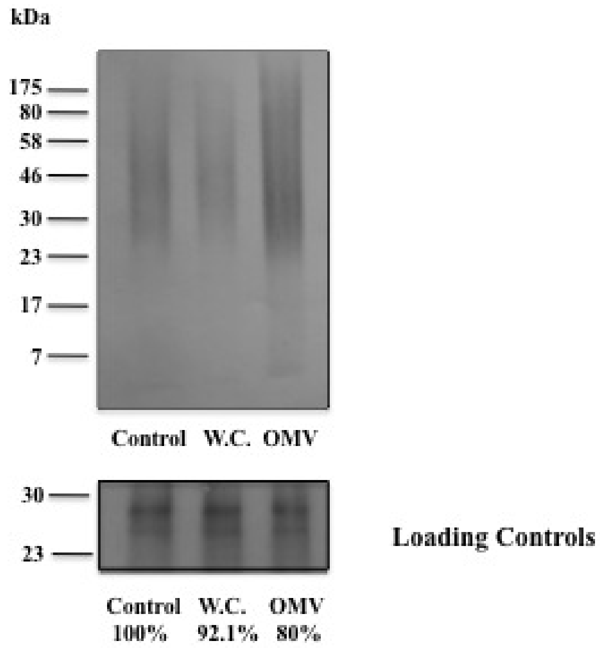

Figure S3. **Titration of A-LPS in *P. gingivalis* W50 whole cells and OMVs versus MAb 1B5.** Whole cell extracts (from 0.8 μg of cells) and OMVs (0.8 μg) were subjected to SDS-PAGE and Western blotting versus MAb 1B5 (upper panel). The lower panel contains whole cell extracts (4 μg of cells) and OMVs (4 μg) of *P. gingivalis* W50 subjected to SDS-PAGE and Western blotting versus MAb 1A1.

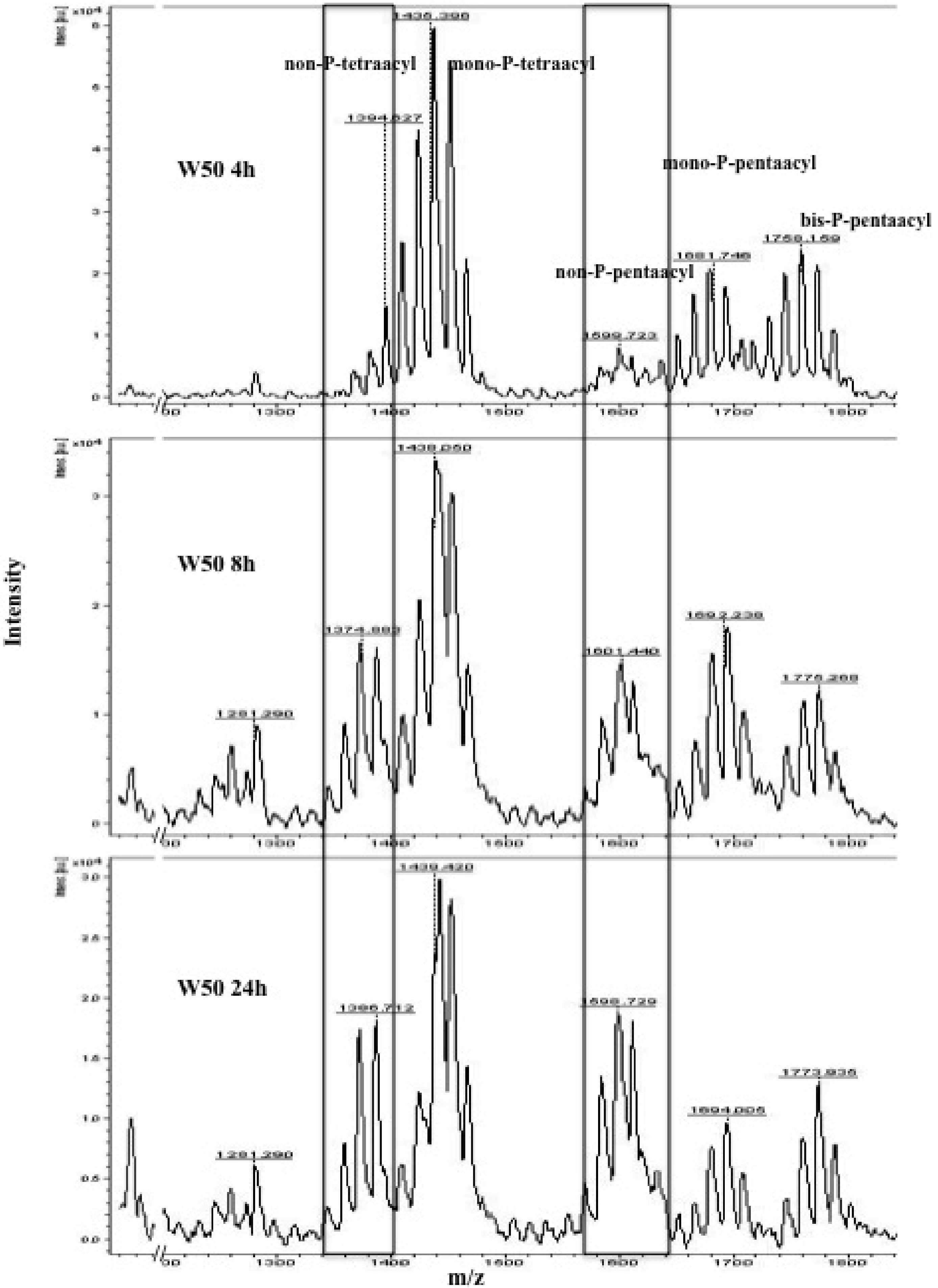

Figure S4. MALDI-TOF MS analysis of lipid A from LPS of *P. gingivalis* W50 strain during growth in BHI.

Table S1A. Strains used.

P. gingivalis strain	Method of manipulation		Reference
W50	Wild type, ATCC 53		
PG0027	Insertion –deletion of PG0027; *erm*^{r}		This paper
cPG0027	Complemented with PG0027 carried on an integrative plasmid (pExp232) to the site of inactivated *erm* in PG0027; *tet*^{r}		This paper
PG0902/27	Additional copy of PG0027 (pExp232) integrated at site of inactivated PG0902; *tet*^{r}		This paper
PG1711/27	Additional copy of PG0027 (pExp232) integrated at site of inactivated PG1711; *tet*^{r}		This paper
PG0027R	Additional copy of PG0027, engineered to be expressed from *rgpA* promoter and then integrated into PG0027; *tet*^{r}		This paper
